# Supplementary figures and images for: Effectiveness and cost-effectiveness of Chuna manual therapy for temporomandibular disorder: A randomized clinical trial
Source: PLoS One. 2025 May 7;20(5):e0322402. doi: 10.1371/journal.pone.0322402 (PMC12057850; doi:10.1371/journal.pone.0322402)

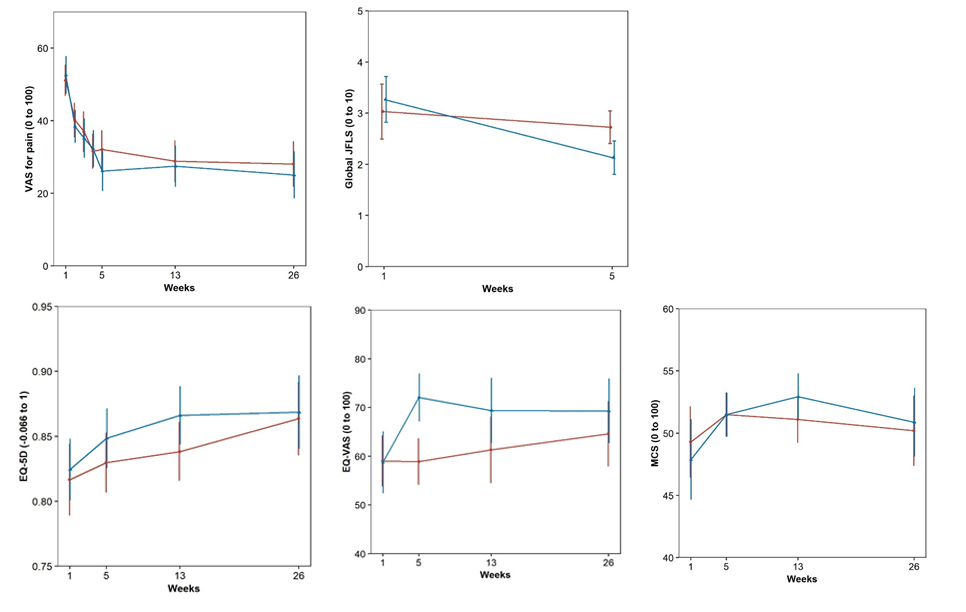

Supplement: S1 Fig — (TIF) [file pone.0322402.s001.tif]

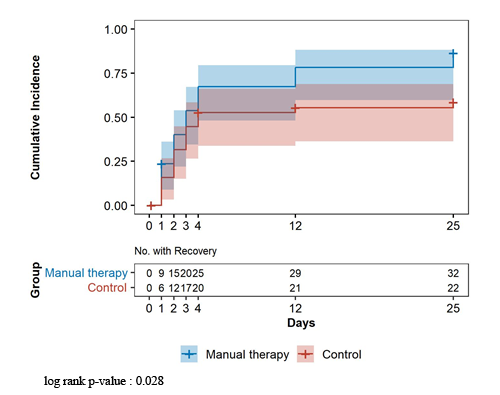

Supplement: S2 Fig — (TIF) [file pone.0322402.s002.tif]
