# Supplementary material for: Effectiveness and cost-effectiveness of Chuna manual therapy for temporomandibular disorder: A randomized clinical trial
Source: PLoS One. 2025 May 7;20(5):e0322402. doi: 10.1371/journal.pone.0322402 (PMC12057850; doi:10.1371/journal.pone.0322402)
Supplement: S1 Table — (DOCX) [file pone.0322402.s003.docx]

S1 Table**.** Cost Calculation Method, Associated Data Sources, and Unit costs

| **Type of costs** | **Calculation method** | **Source of the Original Data** | **Unit cost** |
| --- | --- | --- | --- |
| Manual therapy (*Chuna*) † | The calculation was based on the pricing criteria of the HIRA. *Chuna* manual therapy is divided into simple, complex, and special according to the techniques applied. If there is a correction technique during the procedure, it is classified as complex, and during the intervention period, there was a correction technique in all procedures. | HIRA price index 2019 | $33 |
| Physical therapy  (Electrotherapy and thermotherapy) † | All details of the physical therapy performed on the patient are recorded, and the prices of the techniques corresponding to the HIRA price index are applied. | HIRA price index 2019 | $6  [4–11] |
| Consultation fee at the first visit to a Korean medicine doctor | When a patient visits a medical institution, a medical doctor conducts an examination. The consultation fee is set differently for first and recursive visits, and it also differs between Korean medicine doctors and general practitioners. The manual therapy (*Chuna*) group was consulted by a Korean medicine doctor and the usual care group by a general practitioner. | HIRA price index 2019 | $12 |
| Consultation fee at recursive visits to a Korean medicine doctor‡ |  | HIRA price index 2019 | $8 |
| Consultation fee at the first visit to a Western medicine doctor‡ |  | HIRA price index 2019 | $15 |
| Consultation fee at recursive visits to a Western medicine doctor‡ |  | HIRA price index 2019 | $11 |
| Syndrome differentiation technique fee‡ | Korean traditional medicine has a system that examines comprehensive symptoms based on unique theories and experiences. This is called syndrome differentiation, and all Korean medicine doctors may charge an additional syndrome differentiation technique fee according to the examination. This can be charged once a week, and the patients visited up to twice a week during the five-week intervention period. | HIRA price index 2019 | $3 |
| Radiography† | On the patient’s first visit, a radiographic examination of the temporomandibular joint was performed once. | HIRA price index 2019 | $8 |
| Prescription§ | Through EMR, the type and dose of the drugs and the number of days of prescription drugs were surveyed during the intervention period. In the case of prescription drugs, all the costs were set by HIRA and were applied for each type of prescription drug. Dispensing fees are set by HIRA and are applied according to the number of days of prescription. | HIRA price index 2019 | $9  [4–13] |
| Over the counter¶ | If a patient purchases a prescription drug over the counter, the amount spent is surveyed. | Patient survey | $3  [3–6] |
| Additional private Korean traditional medicine outpatient visits** | The patient’s visits to medical institutions other than clinical trial sites and the associated expenses were surveyed. In this case, the amount indicated in the patient’s response was out-of-pocket payments for health insurance benefit services and non-benefit services, and the benefits were unknown. Accordingly, the benefits were calculated from the claim data of patients with temporomandibular joint disorder in the HIRA-NPS in 2018. The calculated average benefits were stratified by the patient’s sex and age and matched. | Patient survey  2018 HIRA-NPS ^21^ | $25  [22–57] |
| Additional private Western medicine outpatient visits** |  |  | $35  [18–66] |
| Exercise, massage, etc.†† | The cost and number of services purchased by the patient were surveyed. | Patient survey | $21  [0–649] |
| Transportation‡‡ | The transportation cost to visit the clinical trial site was surveyed one week after the baseline, and this was multiplied by the number of visits. | Patient survey | $3  [2–17] |
| Time cost§§ | After one week from the baseline, all the time taken for the patients to leave the house, go to the hospital, be interview, wait, receive treatment, and return home was surveyed. This was multiplied by the number of visits. | Patient survey | — |
| Productivity cost§§ | Productivity loss was assessed using the Work Productivity and Activity Impairment – Specific Health Problem (WPAI-SHP). Then, according to the human capital approach, productivity loss was multiplied by sex and age-stratified income to calculate the income loss due to productivity loss, which is regarded as the productivity costs. In the baseline analysis, overall work impairment was applied to the productivity loss of employed patients, and activity impairment was applied to unemployed patients. The result of estimating the productivity costs only for employed patients is presented in the sensitivity analysis. | Patient survey | — |
| Income§§ | Sex- and age-stratified income was applied. | 2019 survey report on labor conditions by employment type ^22^ | — |

*Abbreviations.* ***HIRA***, Health Insurance Review and Assessment Service; ***EMR,*** Electronic Medical Record; ***HIRA-NPS***, Health Insurance Review and Assessment Service-National Patient Sample; ***WPAI-SHP***, Work Productivity and Activity Impairment – Specific Health Problem.

* When applying a uniform cost, only the unit cost is presented. If the costs are set in a patient-specific manner, the mean [minimum–maximum] of the calculated costs is indicated. All costs were converted to USD as at 2019 (1,156 KRW = 1 USD)

† Price for each examination/treatment session

‡ Price for each visit to the clinical trial institution

§ Price for each prescription

¶ Price for each over-the-counter purchase

** Price for each visit to the medical institution

†† Price for the one-time use of the service

‡‡ Price for each visit to the clinical trial institution

§§ For these costs, the costs incurred by the patients are not directly surveyed, and associated costs are calculated indirectly by considering the patients’ income. Therefore, unit costs are not presented in these cases.
