# Supplementary material for: Effectiveness and cost-effectiveness of Chuna manual therapy for temporomandibular disorder: A randomized clinical trial
Source: PLoS One. 2025 May 7;20(5):e0322402. doi: 10.1371/journal.pone.0322402 (PMC12057850; doi:10.1371/journal.pone.0322402)
Supplement: S2 Table — (DOCX) [file pone.0322402.s004.docx]

S2 Table. List of *Chuna* Treatments Provided to Patients during the Intervention Period

| **Group** | **Method** | **Number of prescribed patients** | **Total prescribed sessions per patient** |
| --- | --- | --- | --- |
| *Chuna* | Sitting TMJ distraction with thumb technique | 40 (100) | 7.4 ± 1.6 |
|  | Sitting lateral pterygoid pushing with index finger technique | 30 (75) | 7.3 ± 1.8 |
|  | Sitting TMJ manipulation with thumb technique | 21 (52.5) | 7.1 ± 2.1 |
|  | Supine cervical spine distraction technique | 30 (75) | 7.3 ± 1.8 |
|  | Supine cervical spine JS distraction manipulation technique | 40 (100) | 7.4 ± 1.6 |
|  | Supine cervical spine manipulation technique | 29 (72.5) | 7.3 ± 1.8 |
| Usual care | Superficial Heat Therapy | 16 (40) | 7.7 ± 0.9 |
|  | Deep Heat Therapy | 24 (60) | 7.3 ± 1.8 |
|  | Interferential Current Therapy | 28 (70) | 7.3 ± 1.8 |
|  | Transcutaneous Electrical Nerve Stimulation | 12 (30) | 7.3± 1.2 |

*Abbreviations*. ***TMJ,*** temporomandibular joint;
