# Supplementary material for: Effectiveness and cost-effectiveness of Chuna manual therapy for temporomandibular disorder: A randomized clinical trial
Source: PLoS One. 2025 May 7;20(5):e0322402. doi: 10.1371/journal.pone.0322402 (PMC12057850; doi:10.1371/journal.pone.0322402)
Supplement: S3 Table — (DOCX) [file pone.0322402.s005.docx]

S3 Table. List of Medication-Related to Temporomandibular Joint Disorders Prescribed to Patients During the Study

|  | | | |  | |
| --- | --- | --- | --- | --- | --- |
|  | ***Chuna* manual therapy (n = 40)** | | **Usual care (n = 40)** | | |
|  | Number of prescribed patients | Total prescribed days per patient | Number of prescribed patients | | Total prescribed days per patient |
| **Total** | 2 (5) | 24.0 ± 32.5 | 7 (17.5) | | 29.3 ± 44.4 |
| Intervention Period | 1 (2.5) | 1 ± NA | 3 (7.5) | | 2 ± 1.7 |
| Acetaminophen | 1 (2.5) | 1 ± NA | 3 (7.5) | | 2 ± 1.7 |
| Follow-up Period | 1 (2.5) | 47 ± NA | 5 (12.5) | | 39.8 ± 49.7 |
| Aceclofenac | 1 (2.5) | 14 ± NA | — | | — |
| Acetaminophen + Caffeine Anhydrous + Isopropylantipyrine | — | — | 1 (2.5) | | 5 ± NA |
| Acetaminophen + Chlorzoxazone | — | — | 1 (2.5) | | 62 ± NA |
| Acetaminophen + Tramadol Hydrochloride | — | — | 1 (2.5) | | 31 ± NA |
| Afloqualone | 1 (2.5) | 14 ± NA | — | | — |
| Diazepam | 1 (2.5) | 14 ± NA | — | | — |
| Eperinsone | — | — | 1 (2.5) | | 19 ± NA |
| Etodolac | — | — | 1 (2.5) | | 19 ± NA |
| Ibuprofen + Pamabrom | — | — | 1 (2.5) | | 1 ± NA |
| Methylprednisolone | 1 (2.5) | 14 ± NA | — | | — |
| Naproxen | — | — | 1 (2.5) | | 62 ± NA |
