# Supplementary material for: Effectiveness and cost-effectiveness of Chuna manual therapy for temporomandibular disorder: A randomized clinical trial
Source: PLoS One. 2025 May 7;20(5):e0322402. doi: 10.1371/journal.pone.0322402 (PMC12057850; doi:10.1371/journal.pone.0322402)
Supplement: S4 Table — (DOCX) [file pone.0322402.s006.docx]

| S4 Table. Secondary Outcomes by Treatment and Time Since Randomization | | | | |  | |  |  |  |
| --- | --- | --- | --- | --- | --- | --- | --- | --- | --- |
|  | ***Chuna* manual therapy** | **Usual care** | **Difference in decrease (95% CI) ^a^** |  | | ***P* Value** | | |  |
| Week 5 post-randomization | | | | |  | | | | |
| **ROM of TMJ** |  |  |  | |  | | | | |
| Protrusion | 4.68 (4.11 to 5.24) | 4.25 (3.69 to 4.81) | -0.43 (-1.25 to 0.39) | | .299 | | | | |
| Deviation | 0.89 (0.64 to 1.13) | 0.95 (0.71 to 1.19) | 0.07 (-0.28 to 0.42) | | .701 | | | | |
| Lateral movement of the mandible to right | 11.04 (9.99 to 12.09) | 9.89 (8.84 to 10.94) | -1.15 (-2.68 to 0.38) | | .139 | | | | |
| Lateral movement of the mandible to left | 10.04 (8.99 to 11.09) | 9.37 (8.31 to 10.43) | -0.67 (-2.19 to 0.85) | | .383 | | | | |
| **WPAI-SHP (%)** | 26.31 (21.24 to 31.39) | 32.37 (27.40 to 37.34) | 6.06 (-1.12 to 13.23) | | .097 | | | | |
| Week 13 post-randomization | | | | |  | | | | |
| **ROM of TMJ** |  |  |  | |  | | | | |
| Protrusion | 4.84 (4.10 to 5.58) | 4.60 (3.86 to 5.34) | -0.24 (-1.31 to 0.83) | | .657 | | | | |
| Deviation | 0.63 (0.40 to 0.86) | 0.96 (0.73 to 1.19) | 0.33 (-0.01 to 0.66) | | .054 | | | | |
| Lateral movement of the mandible to right | 9.62 (8.69 to 10.55) | 9.72 (8.81 to 10.63) | 0.10 (-1.23 to 1.43) | | .881 | | | | |
| Lateral movement of the mandible to left | 9.85 (8.94 to 10.76) | 9.08 (8.17 to 9.99) | -0.77 (-2.07 to 0.52) | | .238 | | | | |
| **WPAI-SHP (%)** | 28.80 (22.61 to 34.99) | 30.81 (24.55 to 37.08) | 2.01 (-6.84 to 10.87) | | .651 | | | | |
| Week 25 post-randomization | | | | |  | | |  | |
| **ROM of TMJ** |  |  |  | |  | | | | |
| Protrusion | 5.02 (4.28 to 5.75) | 4.37 (3.61 to 5.14) | -0.64 (-1.70 to 0.41) | | .229 | | | | |
| Deviation | 0.78 (0.56 to 1.01) | 0.94 (0.72 to 1.16) | 0.16 (-0.17 to 0.48) | | .337 | | | | |
| Lateral movement of the mandible to right | 9.85 (8.94 to 10.76) | 9.08 (8.17 to 9.99) | -0.77 (-2.07 to 0.52) | | .238 | | | | |
| Lateral movement of the mandible to left | 10.19 (9.19 to 11.19) | 9.22 (8.23 to 10.21) | -0.97 (-2.40 to 0.45) | | .177 | | | | |
| **WPAI-SHP (%)** | 28.20 (21.68 to 34.73) | 29.04 (22.22 to 35.85) | 0.83 (-8.22 to 9.89) | | .855 | | | | |
| Abbreviations: ***CI***, confidence interval; ***ROM***, range of movement; ***TMJ***, temporomandibular joint; ***WPAI-SHP***, Work Productivity and Activity Impairment Questionnaire: Specific Health Problem. The estimates for each of the groups and differences in the decrease between the two groups at each time point are displayed, together with their 95% CI. *P* Values are indicated alongside the estimated differences, as follows: **P* < .05 | | | | |  | | | | |
