# Supplementary material for: Effectiveness and cost-effectiveness of Chuna manual therapy for temporomandibular disorder: A randomized clinical trial
Source: PLoS One. 2025 May 7;20(5):e0322402. doi: 10.1371/journal.pone.0322402 (PMC12057850; doi:10.1371/journal.pone.0322402)
Supplement: S5 Table — (DOCX) [file pone.0322402.s007.docx]

| S5 Table. Primary and Secondary Outcomes Per Protocol Analysis with Multiple Imputations | | | | |
| --- | --- | --- | --- | --- |
|  | ***Chuna* manual therapy** | **Usual care** | **Difference in decrease**  **(95% CI) ^a^** | ***P* Value** |
| Week 5 post-randomization | | | |  |
| VAS |  |  |  |  |
| Pain | 25.45 (20.41 to 30.49) | 31.37 (26.40 to 36.35) | 5.92 (-1.28 to 13.13) | .106 |
| NRS |  |  |  |  |
| Pain | 2.80 (2.25 to 3.34) | 3.28 (2.74 to 3.81) | 0.48 (-0.30 to 1.26) | .221 |
| Bothersomeness | 2.90 (2.31 to 3.48) | 3.44 (2.86 to 4.02) | 0.54 (-0.30 to 1.39) | .201 |
| EQ-5D-5L | 0.85 (0.83 to 0.87) | 0.83 (0.81 to 0.86) | -0.02 (-0.05 to 0.01) | .255 |
| EQ-VAS | 72.42 (67.64 to 77.20) | 59.14 (54.43 to 63.86) | -13.28 (-20.10 to -6.45)*** | <.001 |
| SF-12 |  |  |  |  |
| PCS | 49.69 (48.02 to 51.36) | 46.14 (44.49 to 47.79) | -3.55 (-5.94 to -1.16)** | .004 |
| MCS | 51.75 (50.06 to 53.45) | 51.53 (49.85 to 53.20) | -0.23 (-2.65 to 2.20) | .854 |
| PGIC‡ | 2.46 (2.20 to 2.72) | 3.03 (2.77 to 3.28) | -0.57 (-0.93 to -0.20)** | .003 |
| JFLS - Global | 2.11 (1.80 to 2.43) | 2.75 (2.44 to 3.07) | 0.64 (0.19 to 1.09)** | .006 |
| JFLS - Mastication | 2.99 (2.62 to 3.36) | 3.59 (3.22 to 3.96) | 0.60 (0.06 to 1.14)** | .029 |
| JFLS - Mobility | 2.19 (1.78 to 2.60) | 2.56 (2.15 to 2.96) | 0.37 (-0.21 to 0.96) | .211 |
| JFLS - Verbal and emotional | 1.18 (0.79 to 1.56) | 2.10 (1.72 to 2.48) | 0.92 (0.37 to 1.47)** | .001 |
| K-BDI II | 7.46 (6.18 to 8.75) | 9.23 (7.97 to 10.50) | 1.77 (-0.07 to 3.60) | .059 |
| ROM of TMJ |  |  |  |  |
| Protrusion | 4.60 (4.03 to 5.17) | 4.23 (3.67 to 4.79) | -0.37 (-1.19 to 0.45) | 371 |
| Deviation | 0.89 (0.65 to 1.12) | 0.93 (0.70 to 1.16) | 0.04 (-0.30 to 0.38) | .81 |
| Lateral movement of the mandible to right | 10.93 (9.86 to 11.99) | 9.83 (8.78 to 10.88) | -1.09 (-2.61 to 0.43) | .156 |
| Lateral movement of the mandible to left | 9.98 (8.90 to 11.05) | 9.37 (8.30 to 10.43) | -0.61 (-2.15 to 0.93) | .432 |
| WPAI-SHP (%) | 26.15 (21.38 to 30.92) | 32.22 (27.52 to 36.93) | 6.08 (-0.75 to 12.90) | .08 |
| Maximum mouth opening without pain | 43.89 (42.34 to 45.44) | 43.79 (42.26 to 45.32) | -0.10 (-2.33 to 2.13) | .929 |
| Week 13 post-randomization | | | |  |
| VAS |  |  |  |  |
| Pain | 26.46 (20.76 to 32.16) | 28.10 (22.41 to 33.78) | 1.64 (-6.55 to 9.82) | .691 |
| NRS |  |  |  |  |
| Pain | 2.92 (2.32 to 3.52) | 3.01 (2.41 to 3.60) | 0.09 (-0.78 to 0.95) | .84 |
| Bothersomeness | 3.16 (2.48 to 3.84) | 3.23 (2.55 to 3.91) | 0.06 (-0.92 to 1.05) | .9 |
| EQ-5D-5L | 0.87 (0.84 to 0.89) | 0.84 (0.82 to 0.86) | -0.03 (-0.06 to 0.01) | .106 |
| EQ-VAS | 69.68 (63.03 to 76.32) | 61.36 (54.74 to 67.99) | -8.32 (-17.87 to 1.24) | .087 |
| SF-12 |  |  |  |  |
| PCS | 50.33 (48.65 to 52.00) | 47.71 (46.04 to 49.38) | -2.62 (-5.03 to -0.21)* | .034 |
| MCS | 53.26 (51.46 to 55.06) | 51.25 (49.46 to 53.04) | -2.01 (-4.60 to 0.58) | .125 |
| PGIC‡ | 2.73 (2.42 to 3.04) | 3.23 (2.93 to 3.53) | -0.50 (-0.94 to -0.06)* | .026 |
| ROM of TMJ |  |  |  |  |
| Protrusion | 4.79 (4.04 to 5.55) | 4.54 (3.80 to 5.28) | -0.25 (-1.33 to 0.83) | .641 |
| Deviation | 0.66 (0.43 to 0.89) | 0.95 (0.72 to 1.17) | 0.29 (-0.04 to 0.61) | .081 |
| Lateral movement of the mandible to right | 9.53 (8.58 to 10.48) | 9.72 (8.78 to 10.65) | 0.18 (-1.18 to 1.55) | .788 |
| Lateral movement of the mandible to left | 9.70 (8.69 to 10.70) | 9.52 (8.53 to 10.51) | -0.18 (-1.62 to 1.26) | .806 |
| WPAI-SHP (%) | 27.74 (21.61 to 33.86) | 30.28 (24.13 to 36.44) | 2.55 (-6.31 to 11.41) | .568 |
| Maximum mouth opening without pain | 44.08 (42.46 to 45.69) | 43.76 (42.17 to 45.34) | -0.32 (-2.64 to 2.00) | .785 |
| Week 26 post-randomization | | | |  |
| VAS |  |  |  |  |
| Pain | 23.96 (17.71 to 30.21) | 27.42 (21.22 to 33.62) | 3.46 (-5.49 to 12.40) | .443 |
| NRS |  |  |  |  |
| Pain | 2.75 (2.10 to 3.40) | 2.71 (2.07 to 3.36) | -0.04 (-0.97 to 0.89) | .937 |
| Bothersomeness | 3.11 (2.37 to 3.84) | 3.18 (2.44 to 3.91) | 0.07 (-0.99 to 1.13) | .892 |
| EQ-5D-5L | 0.87 (0.84 to 0.90) | 0.87 (0.84 to 0.89) | -0.01 (-0.05 to 0.03) | .754 |
| EQ-VAS | 69.96 (63.50 to 76.42) | 64.86 (58.35 to 71.38) | -5.10 (-14.44 to 4.24) | .28 |
| SF-12 |  |  |  |  |
| PCS | 49.73 (47.80 to 51.66) | 49.43 (47.42 to 51.44) | -0.30 (-3.15 to 2.54) | .832 |
| MCS | 51.15 (48.40 to 53.90) | 50.28 (47.50 to 53.05) | -0.87 (-4.86 to 3.11) | .664 |
| PGIC‡ | 2.90 (2.57 to 3.24) | 3.26 (2.92 to 3.59) | -0.36 (-0.84 to 0.13) | .146 |
| K-BDI II | 6.97 (5.46 to 8.49) | 9.29 (7.77 to 10.81) | 2.32 (0.14 to 4.50)* | .038 |
| ROM of TMJ |  |  |  |  |
| Protrusion | 4.98 (4.23 to 5.72) | 4.33 (3.59 to 5.08) | -0.64 (-1.71 to 0.43) | .234 |
| Deviation | 0.76 (0.55 to 0.98) | 0.92 (0.70 to 1.14) | 0.15 (-0.16 to 0.47) | .336 |
| Lateral movement of the mandible to right | 9.78 (8.86 to 10.70) | 9.08 (8.17 to 9.99) | -0.70 (-2.02 to 0.61) | .289 |
| Lateral movement of the mandible to left | 10.18 (9.16 to 11.21) | 9.19 (8.17 to 10.20) | -1.00 (-2.48 to 0.48) | .181 |
| WPAI-SHP (%) |  |  |  |  |
| Maximum mouth opening without pain | 43.90 (42.36 to 45.44) | 43.20 (41.68 to 44.71) | -0.70 (-2.92 to 1.51) | .528 |
| Abbreviations: ***CI***, confidence interval; ***VAS***, visual analog scale; ***NRS***, numeric rating scale; ***JFLS***, jaw functional limitation scale; ***EQ-5D***-***5L,*** the EuroQol 5 Dimension 5-level; ***SF-12,*** the Medical Outcomes Study 12-Item Short-Form Health Survey; ***PCS,*** Physical Component Summary; ***MCS***, Mental Component Summary; ***PGIC***, Patient Global Impression of Change; ***EQ-VAS***, EuroQol-5 dimension visual analog scale; ***K-BDI II,*** Korean version of Beck’s depression index-2; ***ROM***, range of movement; ***TMJ***, temporomandibular joint; ***WPAI-SHP***, Work Productivity and Activity Impairment Questionnaire: Specific Health Problem  ‡***PGIC*** was assessed on a scale from 1 (“improved”) to 7 (“worsened”), with a lower score indicating more improvement. The difference between the two groups was analyzed using the independent t-test on the endpoint values.  ^a^ The effectiveness outcomes were assessed as the decrease since baseline, and the differences between the two groups were analyzed using analysis of covariance, adjusting for the baseline value, except for PGIC. The primary endpoint was the outcome at **week 5 post-randomization**. *P* Values are indicated alongside the estimated differences, as follows: **P* < .05; ***P* < .01; ****P* < .001. | | | | |
