# Supplementary material for: Effectiveness and cost-effectiveness of Chuna manual therapy for temporomandibular disorder: A randomized clinical trial
Source: PLoS One. 2025 May 7;20(5):e0322402. doi: 10.1371/journal.pone.0322402 (PMC12057850; doi:10.1371/journal.pone.0322402)
Supplement: S6 Table — (DOCX) [file pone.0322402.s008.docx]

| S6 Table. Area Under the Curve of Outcomes According to Treatment | | | | |
| --- | --- | --- | --- | --- |
|  | **Chuna manual therapy** | **Usual care** | **Difference (95% CI)** | ***P* Value** |
| VAS | 700.39 (592.84 to 807.95) | 764.00 (656.28 to 871.73) | -63.61 (-218.42 to 91.20) | .415 |
| NRS for pain | 76.32 (64.40 to 88.24) | 79.92 (68.01 to 91.83) | -3.60 (-20.80 to 13.60) | .678 |
| NRS for bothersomeness | 84.16 (71.08 to 97.24) | 85.62 (72.46 to 98.77) | -1.45 (-20.41 to 17.51) | .879 |
| JFLS - Mastication | 14.72 (13.88 to 15.55) | 15.81 (15.00 to 16.61) | -1.09 (-2.29 to 0.10) | .072 |
| JFLS - Mobility | 10.13 (9.27 to 11.00) | 10.83 (9.98 to 11.67) | -0.69 (-1.87 to 0.49) | .248 |
| JFLS - Verbal and emotional | 6.86 (6.06 to 7.67) | 8.58 (7.79 to 9.37) | -1.72 (-2.87 to -0.56) | .004* |
| JFLS - Global | 10.56 (9.91 to 11.22) | 11.75 (11.11 to 12.38) | -1.18 (-2.10 to -0.26) | .013* |
| K-BDI II | 194.41 (165.33 to 223.49) | 237.72 (208.59 to 266.86) | -43.31 (-85.88 to -0.75) | .046* |
| EQ-5D-5L score | 21.47 (21.06 to 21.89) | 21.03 (20.62 to 21.45) | 0.44 (-0.15 to 1.03) | .142 |
| EQ-VAS | 1729.68 (1626.91 to 1832.45) | 1534.88 (1430.11 to 1639.65) | 194.80 (43.41 to 346.20) | .012* |
| PCS | 1239.70 (1209.14 to 1270.25) | 1191.07 (1160.16 to 1221.99) | 48.62 (4.42 to 92.82) | .032* |
| MCS | 1292.49 (1256.83 to 1328.16) | 1268.83 (1233.06 to 1304.60) | 23.66 (-27.39 to 74.72) | .358 |
| WPAI-SHP | 725.58 (609.95 to 841.21) | 785.32 (669.86 to 900.79) | -59.74 (-225.90 to 106.42) | .476 |

Abbreviations: ***CI***, confidence interval; ***VAS***, visual analog scale; ***NRS***, numeric rating scale; ***JFLS***, jaw functional limitation scale; ***EQ-5D***-***5L,*** EuroQol 5 Dimension 5-level; ***SF-12,*** Medical Outcomes Study 12-Item Short-Form Health Survey; ***PCS,*** Physical Component Summary; ***MCS***, Mental Component Summary; ***EQ-VAS***, EuroQol-5 dimension visual analog scale; ***K-BDI II,*** Korean version of Beck’s depression index-2; ***WPAI-SHP***, Work Productivity and Activity Impairment Questionnaire: Specific Health Problem

*P* Values are indicated alongside the estimated differences as follows: **P* < .05
