# Supplementary material for: Effectiveness and cost-effectiveness of Chuna manual therapy for temporomandibular disorder: A randomized clinical trial
Source: PLoS One. 2025 May 7;20(5):e0322402. doi: 10.1371/journal.pone.0322402 (PMC12057850; doi:10.1371/journal.pone.0322402)
Supplement: S7 Table — (DOCX) [file pone.0322402.s009.docx]

S7 Table. Costs per Patient After Randomization into the *Chuna* Manual Therapy and Usual Care Groups

|  | ***Chuna* manual therapy** | **Usual Care** | **Difference** | ***P* Value** |
| --- | --- | --- | --- | --- |
| **Healthcare system perspectives** | |  |  |  |
| 1st to 5th week | 331 (307 to 349) | 166 (156 to 175) | 165 (140 to 185)* | .002 |
| 1st quarter | 331 (308 to 349) | 180 (163 to 202) | 151 (119 to 176)* | .002 |
| 2nd quarter | 11 (0 to 31) | 11 (0 to 37) | -1 (-27 to 25) | .957 |
| Within trial | 342 (314 to 369) | 192 (164 to 231) | 150 (100 to 193)* | .002 |
| **Non-healthcare cost** | |  |  |  |
| Within trial | 286 (247 to 327) | 276 (240 to 311) | 9 (-44 to 64) | .723 |
| **Healthcare system + Non-healthcare cost** | | |  |  |
| Within trial | 627 (575 to 682) | 468 (418 to 525) | 160 (79 to 230)* | .002 |
| **Productivity loss** | |  |  |  |
| 1st to 5th week | 167 (130 to 204) | 213 (170 to 263) | -47 (-109 to 6) | .096 |
| 1st quarter | 2,221 (1,792 to 2,658) | 2,521 (2,015 to 3,052) | -299 (-972 to 376) | .38 |
| 2nd quarter | 2,278 (1,738 to 2,844) | 2,477 (1,794 to 3,182) | -199 (-1,051 to 631) | .667 |
| Within trial | 4,499 (3,606 to 5,401) | 4,997 (3,936 to 6,264) | -498 (-1,957 to 888) | .482 |
| **Societal perspectives** | |  |  |  |
| 1st to 5th week | 1,416 (1,259 to 1,583) | 1,376 (1,202 to 1,558) | 40 (-199 to 282) | .783 |
| 1st quarter | 2,838 (2,419 to 3,303) | 2,977 (2,468 to 3,610) | -139 (-887 to 561) | .711 |
| 2nd quarter | 2,289 (1,773 to 2,918) | 2,488 (1,812 to 3,233) | -199 (-1,102 to 691) | .713 |
| Within trial | 5,127 (4,241 to 5,992) | 5,465 (4,380 to 6,599) | -338 (-1,801 to 1,035) | .619 |

The confidence interval was estimated with bootstrapping. The 1^st^ quarter to 2^nd^ quarter indicate the interval from baseline to 3 months and 3 to 6 months, respectively. All values are presented as mean and 95% confidence interval. The difference between the two groups was estimated using independent t-test.

* *P* < .05.
