# Supplementary material for: Effectiveness and cost-effectiveness of Chuna manual therapy for temporomandibular disorder: A randomized clinical trial
Source: PLoS One. 2025 May 7;20(5):e0322402. doi: 10.1371/journal.pone.0322402 (PMC12057850; doi:10.1371/journal.pone.0322402)
Supplement: S8 Table — (DOCX) [file pone.0322402.s010.docx]

S8 Table. Sensitivity Analysis with Cost-Effectiveness Analysis for *Chuna* Manual Therapy Compared with Usual Care (EQ-5D-5L)

| **QALY index** | **Sensitivity analysis 1^a^** | | **Sensitivity analysis 2^b^** | **Sensitivity analysis 3^c^** | **Sensitivity analysis 4^d^** | | | | | |
| --- | --- | --- | --- | --- | --- | --- | --- | --- | --- | --- |
|  | **Societal Perspectives** | **Healthcare System Perspectives** | **Healthcare System Perspectives** | **Societal Perspectives** | | **Societal Perspectives** | | **Healthcare System Perspectives** |  |  |
| Difference in QALY | 0.008 (-0.003 to 0.020) | | 0.008  (-0.003 to 0.020) | 0.008  (-0.003 to 0.020) | 0.011 (-0.019 to 0.040) | | | | |  |
| Difference in cost | -499 (-1,893 to 924) | 161 (119 to 194) | 160 (79 to 230) | -1,059 (-2,674 to 371) | -737 (-3,937 to 2,477) | | 149 (53 to 241) | | | |
| ICER ($) | Dominant | 19,133 | 18,953 | Dominant | | Dominant | 13,671 | | | |
| Probability of cost-effectiveness by cost-effectiveness plane (%) |  |  |  |  | |  |  | | | |
| Cost-saving + More effective | 67.9 | — | — | 85.8 | | 51.5 | — | | |  |
| Cost-increasing + More effective | 25.4 | 93.3 | 93.9 | 8.1 | | 25.2 | 76.7 | | | |
| Cost-saving + Less effective | 5.2 | 6.7 | 6.1 | 5.9 | | 15.6 | 0.2 | | | |
| Cost-increasing + Less effective | 1.5 | — | — | 0.2 | | 7.7 | 23.1 | | | |
| Probability of cost-effectiveness at 1xWTP per capita (%) | 80.8 | 65.4 | 65.1 | 95.8 | | 72.2 | 63 | | | |
| Incremental net benefit at 1xWTP per capita ($) | 694 (-712 to 2,207) | 65 (-237 to 372) | 60 (-242 to 358) | 1280 (-181 to 2,904) | | 1,012 (-2,224 to 4,148) | 120 (-623 to 868) | | | |

Abbreviations. ***QALY***, Quality-adjusted life-years; **EQ-5D-5L**, EuroQol 5-Dimension 5-Level; ***ICER***, incremental cost-effectiveness ratio; ***WTP,*** willingness to pay.

* For the baseline analysis, the QALY was calculated using the EQ-5D-5L. The incremental cost was divided by the incremental QALY to calculate the ICER. After nonparametric bootstrapping, the incremental net benefit and probability of cost-effectiveness were calculated using the 1xWTP threshold ($26,375). The costs from the healthcare system perspective include the costs of formal and informal healthcare involved in chronic neck pain treatment and of transportation and time. From a societal perspective, productivity costs from chronic neck pain were included.

^a^ Sensitivity analysis, 1. A per-protocol analysis was performed. The 37 patients in the *Chuna* manual therapy group and 38 in the usual care group were included.

^b^ Sensitivity analysis 2. Non-healthcare costs were also considered from the healthcare system perspective.

^c^ Sensitivity analysis 3. Productivity costs for unemployed patients were regarded as zero.

^d^ Sensitivity analysis 4. It was assumed that the results of the clinical trial at 26 weeks would be maintained for up to one year.
