# Supplementary material for: Effectiveness and cost-effectiveness of Chuna manual therapy for temporomandibular disorder: A randomized clinical trial
Source: PLoS One. 2025 May 7;20(5):e0322402. doi: 10.1371/journal.pone.0322402 (PMC12057850; doi:10.1371/journal.pone.0322402)
Supplement: S9 Table — (DOCX) [file pone.0322402.s011.docx]

S9 Table. Sensitivity Analysis with Cost-Effectiveness Analysis for Chuna Manual Therapy Compared with Usual Care (SF-6D)

| **QALY index** | **Sensitivity analysis 1^a^** | | **Sensitivity analysis 2^b^** | **Sensitivity analysis 3^c^** | **Sensitivity analysis 4^d^** | | | | | |
| --- | --- | --- | --- | --- | --- | --- | --- | --- | --- | --- |
|  | **Societal Perspectives** | **Healthcare System Perspectives** | **Healthcare System Perspectives** | **Societal Perspectives** | | **Societal Perspectives** | | **Healthcare System Perspectives** |  |  |
| Difference in QALY | 0.017 (0.003 to 0.031) | | 0.016 (0.002 to 0.031) | 0.016 (0.002 to 0.031) | 0.029 (-0.006 to 0.063) | | | | |  |
| Difference in cost | -499 (-1,893 to 924) | 161 (119 to 194) | 160 (79 to 230) | -1,059 (-2,674 to 371) | -737 (-3,937 to 2,477) | | 149 (53 to 241) | | | |
| **ICER ($)** | Dominant | 9,768 | 9,675 | Dominant | | Dominant | 5,212 | | | |
| **Probability of cost-effectiveness by cost-effectiveness plane (%)** |  |  |  |  | |  |  | | | |
| Cost-saving + More effective | 73 | — | — | 91.3 | | 63.4 | 0.2 | | |  |
| Cost-increasing + More effective | 26.7 | 99.7 | 99.4 | 8.1 | | 31.8 | 95 | | | |
| Cost-saving + Less effective | 0.1 | 0.3 | 0.6 | 0.4 | | 3.7 | 0 | | | |
| Cost-increasing + Less effective | 0.2 | — | — | 0.2 | | 1.1 | 4.8 | | | |
| **Probability of cost-effectiveness at 1xWTP per capita (%)** | 88.7 | 94.5 | 93.6 | 98 | | 82.1 | 91.2 | | | |
| **Incremental net benefit at 1xWTP per capita ($)** | 929 (-506 to 2,434) | 299 (-63 to 663) | 282 (-85 to 623) | 1,501 (66 to 3,114) | | 1,498 (-1,884 to 4,828) | 606 (-289 to 1,446) | | | |

Abbreviations. ***QALY***, Quality-adjusted life-years; ***SF-6D***, short-form 6-dimension; ***ICER***, incremental cost-effectiveness ratio; ***WTP,*** willingness to pay.

* For the baseline analysis, the QALY was calculated using the SF-6D. The incremental cost was divided by the incremental QALY to calculate the ICER. After nonparametric bootstrapping, the incremental net benefit and probability of cost-effectiveness were calculated using the 1xWTP threshold ($26,375). The costs from the healthcare system perspective include the costs of formal and informal healthcare involved in chronic neck pain treatment and of transportation and time. From a societal perspective, productivity costs from chronic neck pain were included.

^a^ Sensitivity analysis, 1. A per-protocol analysis was performed. The 37 patients in the *Chuna* manual therapy group and 38 in the usual care group were included.

^b^ Sensitivity analysis 2. Non-healthcare costs were also considered from the healthcare system perspective.

^c^ Sensitivity analysis 3. Productivity costs for unemployed patients were regarded as zero.

^d^ Sensitivity analysis 4. It was assumed that the results of the clinical trial at 26 weeks would be maintained for up to one year.
