# Supplementary material for: Effectiveness and cost-effectiveness of Chuna manual therapy for temporomandibular disorder: A randomized clinical trial
Source: PLoS One. 2025 May 7;20(5):e0322402. doi: 10.1371/journal.pone.0322402 (PMC12057850; doi:10.1371/journal.pone.0322402)
Supplement: S10 Table — (DOCX) [file pone.0322402.s012.docx]

S10 Table. Sensitivity Analysis with Cost-Effectiveness Analysis for *Chuna* Manual Therapy Compared with Usual Care (EQ-VAS)

| **QALY index** | **Sensitivity analysis 1^a^** | | | **Sensitivity analysis 2^b^** | **Sensitivity analysis 3^c^** | **Sensitivity analysis 4^d^** | | | | | |
| --- | --- | --- | --- | --- | --- | --- | --- | --- | --- | --- | --- |
|  | **Societal Perspectives** | | **Healthcare System Perspectives** | **Healthcare System Perspectives** | **Societal Perspectives** | | **Societal Perspectives** | | **Healthcare System Perspectives** |  |  |
| Difference in QALY | 0.038 (0.010 to 0.067) | | | 0.037 (0.008 to 0.067) | 0.037 (0.008 to 0.067) | 0.038 (0.010 to 0.067) | | | | |  |
| Difference in cost | -499 (-1,893 to 924) | 161 (119 to 194) | | 160 (79 to 230) | -1,059 (-2,674 to 371) | -737 (-3,937 to 2,477) | | 149 (53 to 241) | | | |
| ICER ($) | Dominant | | 4,299 | 4,259 | Dominant | | Dominant | 2,449 | | | |
| Probability of cost-effectiveness by cost-effectiveness plane (%) |  | |  |  |  | |  |  | | | |
| Cost-saving + More effective | 72.7 | | — | — | 91.4 | | 63.3 | 0.2 | | |  |
| Cost-increasing + More effective | 26.9 | | 99.6 | 99.7 | 8.3 | | 32.4 | 95.5 | | | |
| Cost-saving + Less effective | 0.4 | | 0.4 | 0.3 | 0.3 | | 3.8 | 0 | | | |
| Cost-increasing + Less effective | 0 | | — | — | 0 | | 0.5 | 4.3 | | | |
| Probability of cost-effectiveness at 1xWTP per capita (%) | 96.6 | | 98.5 | 98.3 | 98.9 | | 89.6 | 94.4 | | | |
| Incremental net benefit at 1xWTP per capita ($) | 1,473 (-94 to 3,057) | | 843 (109 to 1,645) | 825 (50 to 1,589) | 2,045 (469 to 3,830) | | 2,311 (-1,065 to 5,840) | 1,419 (-335 to 3,133) | | | |

Abbreviations. ***QALY***, Quality-adjusted life-years; ***EQ-VAS***, EuroQol visual analog scale; ***ICER***, incremental cost-effectiveness ratio; ***WTP,*** willingness to pay.

* For the baseline analysis, the QALY was calculated using the EQ-VAS. The incremental cost was divided by the incremental QALY to calculate the ICER. After nonparametric bootstrapping, the incremental net benefit and probability of cost-effectiveness were calculated using the 1xWTP threshold ($26,375). The costs from the healthcare system perspective include the costs of formal and informal healthcare involved in chronic neck pain treatment and of transportation and time. From a societal perspective, productivity costs from chronic neck pain were included.

^a^ Sensitivity analysis, 1. A per-protocol analysis was performed. The 37 patients in the *Chuna* manual therapy group and 38 in the usual care group were included.

^b^ Sensitivity analysis 2. Non-healthcare costs were also considered from the healthcare system perspective.

^c^ Sensitivity analysis 3. Productivity costs for unemployed patients were regarded as zero.

^d^ Sensitivity analysis 4. It was assumed that the results of the clinical trial at 26 weeks would be maintained for up to one year.
