# Supplementary material for: Effectiveness and cost-effectiveness of Chuna manual therapy for temporomandibular disorder: A randomized clinical trial
Source: PLoS One. 2025 May 7;20(5):e0322402. doi: 10.1371/journal.pone.0322402 (PMC12057850; doi:10.1371/journal.pone.0322402)
Supplement: S1 File — (PDF) [file pone.0322402.s013.pdf]

Protocol of Clinical Trial

(Clinical research of effectiveness and economic feasibility of Chuna manual therapy for temporomandibular disorder: A multicenter randomized controlled trial)

Kyung Hee University Korean Medicine Hospital, Gangdong
Jaseng Korean Medicine Hospital
Daejeon Jaseng Korean Medicine Hospital
Bucheon Jaseng Korean Medicine Hospital
Haeundae Jaseng Korean Medicine Hospital

Timetable

	**Study period**								
**Time point**	**Enrollment**	**Allocation**	**Active Treatment post-allocation**				**Follow-up**		
	**Week -1**	**Week 0 (Baseline)**	**Week 1**	**Week 2**	**Week 3**	**Week 4**	**Week 5**	**Month 3**	**Month 6**
**Visit window**			**±3**	**±3**	**±3**	**±3**	**±3**	**±7**	**±7**
Eligibility screening	○								
Written Informed consent	○								
Vital signs	○		○				○		
Sociodemographic characteristics, medical history (e.g. TMJ pain, medication history)	○								
RDC/TMD test & analysis	○								
Randomized allocation		○							
TMJ X-RAY			○						
Credibility and Expectancy			○						
Treatment in Chuna group (experimental group)			← 2 times/week →						
Treatment in UC group (active control group)			← 2 times/week →						
Symptoms and change in medicine			○	○	○	○	○	○	○
NRS of TMJ pain/ bothersomeness	○		← every visit →				○	○	○
VAS of TMJ pain			○	○	○	○	○	○	○
K-BDI-2			○				○		○
JFLS			○				○		
TMJ Range of Motion (maximum mouth opening, mandibular excursive movement)			○	○	○	○	○	○	○
PGIC							○	○	○
EQ-5D-5L			○				○	○	○
EQ-VAS			○				○	○	○
SF-12			○				○	○	○
Economic evaluation-Medical costs			○				○	○	○
Economic evaluation-Time costs				○					
Economic evaluation-Productivity loss			○	○	○	○	○	○	○
Adverse events			← every visit →				○	○	○

* NRS, VAS K-BDI-2, JFLS, TMJ Range of Motion, PGIC, EQ-5D-5L, EQ-VAS, SF-12, measurements for economic evaluation are conducted during the first visit of the relevant week.
* During the clinical period, visits scheduled for Week 1 to Week 5 are allowed within ± 3 days of the scheduled visit date, and visits scheduled for 3 months and 6 months after the first visit (visit 2) of Week 1 are allowed within ± 7 days of the scheduled visit date.
* Subjects selected during the screening visit will begin visit 2 on the same day or within 3 days.

Summary of Protocol

Trial Code	KH-TMDCMT-2018-04
Title	Clinical research of effectiveness and economic feasibility of Chuna manual therapy for temporomandibular disorder: A multicenter randomized controlled trial
Site	Kyung Hee University Korean Medicine Hospital, Gangdong<br>Jaseng Korean Medicine Hospital<br>Daejeon Jaseng Korean Medicine Hospital<br>Bucheon Jaseng Korean Medicine Hospital<br>Haeundae Jaseng Korean Medicine Hospital
Phase	Investigator-initiated clinical trial
Research Period	18 months from IRB approval date
Participants	Patients with chronic unilateral or bilateral temporomandibular joint (TMJ) and facial pain due to temporomandibular disorders, diagnosed as myofascial TMD by a Korean Medicine Doctor.
Number of participants	A total of 80 participants (considering a dropout rate of 30%), with 40 participants in each group. Patient recruitment will be conducted competitively and will continue until the total number of participants is reached.
Objective	This study aims to evaluate the effectiveness and cost-effectiveness of Chuna manual therapy in patients with chronic temporomandibular joint (TMJ) pain.
Study design	After voluntarily signing the consent form, the study subjects (or their legal representatives) will be assessed for eligibility to participate in the study. Eligible subjects will be randomly assigned to either the experimental group or the control group. The subjects will receive 8 treatments over 4 weeks (both the experimental and control groups) as per their assigned group. Additionally, there are 3 follow-up visits planned over 24 weeks after registration.
Inclusion criteria	1) Unilateral or bilateral TMD<br>2) NRS for TMD pain (more severe side for bilateral TMD) of 4 or above<br>3) Continuous or intermittent TMJ pain lasting more than 3 months<br>4) Diagnosed as myofascial TMD, Axis I: Group 1 in RDC/TMD criteria<br>5) Aged 19–70 years at the signing of the participant agreement form<br>6) Agreement to participate in the clinical trial, and signing of the agreement form
Exclusion criteria	1) The symptoms originated from or were precipitated by a traumatic injury (i.e. traffic accident)<br>2) Patients with TMD but not included in Axis 1: Group I of the RDC/ TMD criteria<br>3) Medical history of surgical operations related to TMJ<br>4) Other diseases that can affect the effectiveness and results of the treatment, such as rheumatoid arthritis, neoplastic disease, cerebrovascular accident, myocardial infarction, etc.<br>5) Current assumption of oral medications that can affect the results of the study, such as steroids, immunosuppressive drugs, psychiatric medications, etc.<br>6)) Assumption of medications that can affect pain severity, such as non-steroidal anti-inflammatory drugs (NSAIDs), during the last week, or treated with CMT within the last 2 weeks, for any reason<br>7) Pregnant, planning to have babies or currently breastfeeding<br>8) Participated in other clinical trial within a month from the date of screening, or planning to take part in other trials during the period of this study<br>9) Other conditions investigators consider unfit to this trial
Outcomes	- Primary outcomes<br>1) Visual Analogue Scale (VAS) for TMJ pain<br>- Secondary outcomes<br>1) Numeric Rating Scale (NRS) for TMJ pain and bothersomeness.<br>2) Range of Motion (ROM) of the TMJ

	3) Beck's Depression Inventory (BDI)<br>4) Jaw Functional Limitation Scale (JFLS)<br>5) Patient Global Impression of Change (PGIC)<br>6) Short Form-12 Health Survey (SF-12), version 2<br>7) 5-Level EuroQol-5 Dimension (EQ-5D-5 L) and EuroQol Visual Analogue Scale (EQ-VAS)<br>8) Credibility and expectancy questionnaire<br>9) Cost data investigation<br>10) Adverse events

1. Title

Clinical research of effectiveness and economic feasibility of Chuna manual medicine for temporomandibular disorder: A multicenter randomized controlled trial

2. Site

Name
Kyung Hee University Korean Medicine Hospital, Gangdong
Jaseng Korean Medicine Hospital
Daejeon Jaseng Korean Medicine Hospital
Bucheon Jaseng Korean Medicine Hospital
Haeundae Jaseng Korean Medicine Hospital

3. Participants

3.1. Participants

Patients with chronic unilateral or bilateral temporomandibular joint (TMJ) and facial pain due to temporomandibular disorders, diagnosed as myofascial TMD by a Korean Medicine Doctor

3.2. Inclusion criteria

1) Unilateral or bilateral TMD
2) NRS for TMD pain (more severe side for bilateral TMD) of 4 or above
3) Continuous or intermittent TMJ pain lasting more than 3 months
4) Diagnosed as myofascial TMD, Axis I: Group 1 in RDC/TMD criteria
5) Aged 19–70 years at the signing of the participant agreement form
6) Agreement to participate in the clinical trial, and signing of the agreement form

3.3. Exclusion criteria

1) The symptoms originated from or were precipitated by a traumatic injury (i.e. traffic accident)
2) Patients with TMD but not included in Axis 1: Group I of the RDC/ TMD criteria
3) Medical history of surgical operations related to TMJ
4) Other diseases that can affect the effectiveness and results of the treatment, such as rheumatoid arthritis, neoplastic disease, cerebrovascular accident, myocardial infarction, etc.
5) Current assumption of oral medications that can affect the results of the study, such as steroids, immunosuppressive drugs, psychiatric medications, etc.
6) Assumption of medications that can affect pain severity, such as non-steroidal anti-inflammatory drugs (NSAIDs), during the last week, or treated with CMT within the last 2 weeks, for any reason
7) Pregnant, planning to have babies or currently breastfeeding
8) Participated in other clinical trial within a month from the date of screening, or planning to take part in other trials during the period of this study
9) Other conditions investigators consider unfit to this trial

3.4. Discontinuation and Withdrawal Criteria

Patients have the right to voluntarily withdraw from participating in the clinical study at any time, regardless of the reason. Additionally, the investigator has the authority to discontinue a patient's participation in the study. Reasons for withdrawal from the clinical study may include, but are not limited to, the following:

- Withdrawal of consent by the study subject
- Medical conditions deemed to jeopardize the safety of the patient if the study continues
- When discontinuation of participation is in the best interest of the patient
- Non-compliance with clinical study regulations by the study subject

Efforts will be made to obtain information on patients who discontinue the clinical study, and the reasons for discontinuation of treatment or observation will be recorded. For study subjects whose participation is discontinued, follow-up may continue with patient consent. However, in the event of withdrawal of consent, follow-up will not be conducted, and patients who discontinue participation in the clinical study will not be replaced.

At any time during the clinical study, if a serious adverse event or an adverse reaction deemed harmful to the

study subject is observed, and if subsequent evaluation of the course and causality of the adverse event with the treatment determines that continuation of treatment is harmful to the study subject, the investigator will permanently discontinue the treatment for the patient in this clinical study.

- Study subjects who discontinue before starting treatment are considered screening failures. Unless medically necessary, all required assessments at the time of discontinuation (screening failures) do not have to be completed.
- Subjects who withdraw early due to adverse events may receive appropriate treatment for the adverse events if necessary. In the case of early withdrawal due to adverse events, the investigator or an investigator designee must continuously evaluate the study subject until the adverse event resolves or is deemed permanent.

Other reasons for discontinuation of the clinical study by the investigator include:

- If a disease that could affect the outcome of the study is discovered in the study subject that was not detected in pre-study screening.
- If there are issues performing medical procedures for temporomandibular joint disease in the study subject.
- If the study subject undergoes surgery, procedures, or treatments with drugs that could affect the study outcome without the direction of the attending physician during the treatment period or follow-up period.
- If the investigator deems that the continuation of the study is inappropriate for any other reason.

3.5. Sample size calculation

Calculation of the number of clinical study subjects: As this study is an investigator-initiated clinical trial, the sample size was determined by referencing existing similar studies and clinical experience..
1) Level of significance, α=0.05(two-tailed test)
2) Type II error (β) is set at 0.2, making the power of the test 80%.
3) There is no existing study comparing Korean Chuna therapy and usual care for TMJ-related conditions in Korea. Therefore, based on clinical experience and manual therapy studies in China 1-3), the effect size for comparing the mean differences in pain VAS between the two groups is assumed to be at least medium (effect size = 0.7).
4) G*Power 3.1.7 is used to calculate the sample size.
Assuming an effect size of 0.7, 34 subjects per group (a total of 68 subjects) are required. However, since the main analysis will be performed using ANCOVA, adjusting for baseline outcome values, and assuming a correlation value of 0.3 between baseline and the primary endpoint, the sample size is calculated as (1 - 0.3 * 0.3) * 68, resulting in 62 subjects. Considering a 30% dropout rate during the study, a total of 80 subjects will be recruited, with 40 subjects per group.

4. Research Period

18 months from IRB approval date

5. Method

5.1. Design

The study design is a 2-arm parallel multicenter RCT (Kyung Hee University Hospital of Korean Medicine, Gangdong, Jaseng Hospital of Korean Medicine (Gangnam), Daejeon Jaseng Hospital of Korean Medicine, Bucheon Jaseng Hospital of Korean Medicine, Haeundae Jaseng Hospital of Korean Medicine). Study participants will receive outpatient treatment for a total of 4 weeks. After screening at the initial visit, participants who meet the inclusion/exclusion criteria will be randomly assigned to either the Chuna manual therapy group (40 participants) or the usual care group (40 participants). Treatment for temporomandibular joint (TMJ) pain disorders will be administered according to the developed clinical study protocol.
For the economic evaluation study, cost data collection and utility assessment for estimating QALY (Quality Adjusted Life Year) will be conducted. During the clinical study period, quality of life will be assessed using EQ-5D, EQ-VAS, and SF-12 measurement tools at Visits 2, 10, and 12. Various costs will be assessed using developed survey items at the first visit of each week. Additionally, a separate follow-up survey will be conducted until 6 months (Visit 12) after clinical study enrollment. The survey will be conducted through face-to-face interviews, telephone interviews, or distributed questionnaires, measuring healthcare utilization and quality of life.

5.2. Random allocation and blinding

5.2.1. Random allocation

When subjects who have voluntarily signed the informed consent form are reviewed and determined to meet the inclusion and exclusion criteria, and thus are deemed suitable for the clinical study, they will be assigned to one

of two groups using a block randomization method. This method will be implemented by a statistician using nQuery Advisor 7.0 (or SAS 9.0 or SPSS 21.0), with an equal number of patients (40 each) allocated to both groups. The block size will remain undisclosed to the personnel conducting patient recruitment and group allocation.
Random assignment will occur based on patient registration at each of the five institutions, without stratification by institution. Patient recruitment will follow a competitive enrollment process at each hospital and will continue until the total number of patients is achieved. Participants will be assigned an identification code (Randomized Number) in the order of recruitment according to the random allocation details. The results of the central randomization will be communicated to each institution conducting the clinical study, and treatment will proceed accordingly in each group.

5.2.2. Allocation concealment and blinding
After providing patients with a thorough explanation of the clinical study, those who meet the inclusion and exclusion criteria and sign the written consent form will be assigned to groups using opaque, sealed envelopes. Since it is not feasible to blind the practitioners and participants in this study, only the assessors will be blinded. Assessor, who do not participate in the procedures and are blinded to group assignments, will conduct assessments in a separate area after the procedures are completed. These evaluations will be performed by clinical research coordinator or residents.

5.3. Pre-Clinical Study Evaluation
5.3.1. Patient Consent, Screening Number Assignment, and Demographic Survey
Before the clinical study begins, the purpose and details of the study will be thoroughly explained to the subjects. Written consent will be obtained, and screening numbers will be assigned in the order of receiving the written consents. Afterward, demographic information will be collected.
5.3.2. Medical History and Medication History Investigation
The subject's medical history and medication history will be thoroughly investigated and recorded through interviews and review of past medical records. The details to be included are as follows:
- Surgical history
- Past medical history and comorbidities, treatment history for heart disease and gastrointestinal diseases
- Any adverse reactions to previous pharmacopuncture treatment and general treatments (medication, physical therapy)
- Current medical history
- Evaluation of inclusion/exclusion criteria compliance
- Other medication treatments and treatment history
- Pre-clinical study cost investigation: Investigate the treatment costs related to the disease incurred before participating in the clinical study since the onset of the disease
- Quality of life measurement: Measure the patient's quality of life before participating in the clinical study using tools such as EQ-5D-5L.
5.3.3. RDC/TMD Examination
- The RDC/TMD (Research Diagnostic Criteria for Temporo-Mandibular Disorders) diagnostic tool was proposed by Samuel F. Dworkin and Linda LeResche in 1992 and has been widely used in studies as a diagnostic tool for temporomandibular disorders. According to this tool, temporomandibular disorders can be classified into three categories: myofascial pain, disc displacement, and joint problems. Pain disorders fall into the myofascial pain group and part of the joint problem group. Additionally, the diagnostic and examination process includes a multi-faceted analysis of temporomandibular joint issues, such as mouth opening range, active and passive ROM, degree of pain and discomfort, and other symptoms.
- Moreover, there is a translated version of RDC/TMD by Korean researchers, approved by the international RDC/TMD consortium as validated data suitable for research purposes. In this study, patients confirmed to have temporomandibular joint problems with pain after the RDC/TMD examination will be targeted. The RDC/TMD diagnostic criteria consist of two axes. Axis I, which classifies symptoms, includes three groups: Group I, II, and III. Of these, Groups I and III use pain as an indicator. Group I a/b includes myofascial pain (without mouth opening limitation, a; with mouth opening limitation, b), and Group III a/b includes arthralgia and osteoarthritis, respectively.

5.4. Intervention
5.4.1. Chuna manual therapy

1) Standardization of CMT for treatment of TMD patients.
CMT will be standardized according to the content of this protocol to remove any bias due to the techniques used

and the intensity of the performance.
2) Preparation for the procedure
The participant is sitting on a chair or lying on a table. A KMD washes hands with cleansing.
3) CMT procedure
CMT techniques: 6 techniques are selected as follows by referring to the textbook of Chuna manual medicine [11] and literature on the effect of manual therapy on the cervical spine for TMD patients. The intensity of the distraction technique (two of the techniques for cervical spine) will be set not to make participants feel uncomfortable, but to mobilize them as far as the limits of the passive range of motion. After conducting all of the techniques, the participants will spend 10 min at rest, to observe any adverse events.
4) Frequency and duration of CMT 10) Frequency: a total of eight times over 4 weeks (two times a week) 11) Time duration of a performance: 20 min total (10 min for CMT, and 10 min for rest after treatment)

CMT for TMJ	Sitting TMJ distraction with thumb technique
	Sitting lateral pterygoid pushing with index finger technique
	Sitting TMJ manipulation with thumb technique
CMT for cervical spine	Supine cervical spine distraction technique
	Supine cervical spine JS distraction manipulation technique
	Supine cervical spine manipulation technique

5.4.2. Usual care
- Physical therapies that were proven to alleviate TMD symptoms include thermotherapy, ultrasound therapy, TENS, ICT, etc. In this study, it will be limited to one of thermotherapy, ultrasound therapy, TENS or ICT for the UC group, and will be applied to the area around the neck or the TMJ. Reduction of pain, anti-inflammation, muscle activation, healing, and regeneration of tissue are the mechanisms reported thus far for these therapies [21].
- Frequency and duration of UC: a total of eight times over 4 weeks (two times a week), total 20 min (10 min for the treatment and 10 min for rest after treatment)

5.4.3. Education
- Education on TMD will be conducted for the participants of both groups. It covers the causes, prevention, treatment, management, and self-stretching for TMD, using specially assembled materials.

5.5. Items to be investigated and Methods
5.5.1. Demographic Data / Other Pre-Treatment Characteristics
For patients who meet the inclusion criteria, the following data will be collected:

- Gender
- Date of birth
- Age (in full years)
- Height, weight
- Vital signs: body temperature, blood pressure, pulse rate
- Medical history: During the first visit, the patient's medical history will be thoroughly investigated and recorded through a questionnaire and review of past medical records. This should include past illnesses and comorbidities, history of heart and gastrointestinal diseases, surgical history, current illnesses, and any other medications or treatments received.
- Physical examination: Significant findings from the first visit's examination will be recorded in the physical examination section of the case report form. If any significant physical findings meeting the definition of adverse events are discovered after the start of the study procedure, they will be recorded in the adverse event case report form. However, any undesirable physical symptoms identified before the start of the study procedure should be added to the current illness investigation section.
-

5.5.2. Concomitant Therapy
During the treatment period (week 1-4), medications and treatments from other medical institutions for temporomandibular joint (TMJ) disorders are not allowed. These treatments are permitted during the observation period. Any treatments received during the follow-up period should be recorded at the final visit.

5.5.3. Prohibited Medications
- From study registration until the primary endpoint at week 5 (visit 10), all direct treatments aimed at relieving TMJ pain (e.g., medications, surgeries, other types of acupuncture, and physical therapy) are prohibited for both the clinical study group and the control group. No treatment restrictions are applied during the observation period after the primary endpoint at week 5.

However, during the study period, rescue medication such as acetaminophen (e.g., Tylenol, Acetaminophen tab) will be allowed for all clinical study subjects up to a maximum of 4g per day, and the usage of this medication will be recorded through self-reporting.

5.5.4. Subject Management During the Follow-Up Period After Treatment Completion

- Clinical study subjects who do not feel completely cured by the final visit or those who experience a recurrence of pain after the treatment completion visit will be allowed to receive treatment at a medical institution of their choice. The details of such treatments will be recorded during the follow-up visits.
- In preparation for any unexpected delayed adverse reactions, subjects will be allowed to seek medical care at any time under the researcher's instructions.

5.6. Imaging Examination Items

- The X-ray examination
- Anteroposterior (closed) and both lateral views (closed, open) X-rays will be performed.

6. Outcomes

6.1. Primary outcomes

Visual Analogue Scale (VAS) for TMJ pain

VAS will be evaluated on the 1st, 3rd, 5th, and 7th treatment visits (week 1–4) and 5 weeks, 3 months, and 6 months after allocation. The average TMJ pain for the past week will be investigated. The primary endpoint is VAS at 5 weeks after allocation (a week after the last treatment). The far left end of a 100 mm scale line means "no pain" and the far right end means "the worst pain imaginable." It is the most frequently used way to measure the pain severity. A participant puts a mark on the line, which approximates his/her own pain for the past week. If a participant complains of different pain levels on each TMJ, the more severe side will be recorded.

6.2. Secondary outcomes

Numeric Rating Scale (NRS) for TMJ pain and bothersomeness.

This outcome will be checked at screening, at every treatment visit, and 5 weeks, 3 months, and 6 months after allocation. Participants will be asked to select one digit that shows the level of TMJ pain and bothersomeness for the past week (0 means no pain or bothersomeness, and 10 the most severe degree).

Range of Motion (ROM) of the TMJ

The ROM of the TMJ will be evaluated at screening, and at the 1st, 3rd, 5th, 7th treatment visits (week 1–4) and after 5 weeks. Active maximum mouth opening without pain, protrusion, and each lateral movement of the mandible will be measured using the Range of Motion Ruler (Therabite, Sweden). Participants will be asked to open the mouth as much as possible without any pain, to slightly open the mouth and protrude the mandible as much as possible and to slightly open the mouth and move the jaw as much as possible to each side. The measuring process will be concretely conducted in accordance with the specifications for clinical examination provided by the international RDC/TMD consortium.

Beck's Depression Inventory (BDI).

This questionnaire will be conducted on the 1st treatment visit, before the treatment, 1 week after the last treatment, and at the 6-month follow-up. It is composed of 21 questions, and a value from 0 to 3 points is assigned to each answer. A higher score means a more severe depressive state. Clinical depressive disorder is diagnosed when the total score is 17 or above. In this case, the investigators will invite the participant to visit a psychological clinic.

Jaw Functional Limitation Scale (JFLS).

Participants will be asked to complete this questionnaire on the 1st treatment visit, prior to the treatment, and a week after the last treatment. The functions of biting, moving, speaking, and emotional expression will be investigated through 20 items. Each item can be rated in the answer from 0 (no limitations) to 10 (maximum limitation).

Patient Global Impression of Change (PGIC).

The PGIC questionnaire will be administered a week after the last treatment, and at the 3-month and 6-month follow-ups. Participants will subjectively evaluate the improvement of their health state with a 7- point Likert scale, ranging from 1 (very much improved) to 7 (very much worse).

Short Form-12 Health Survey (SF-12), version 2.

SF-12 will be conducted on the 1st treatment visit, prior to the treatment, a week after the last treatment, and at the 3-month and 6-month follow-ups. It contains 12 items about health-related quality of life. A higher total score reflects a better quality of life.

5-Level EuroQol-5 Dimension (EQ-5D-5 L) and EuroQol Visual Analogue Scale (EQ-VAS).

EQ-5D-5 L and EQ-VAS will be conducted on the 1st treatment visit, just before the treatment, a week after the last treatment, and at the 3-month and 6-month follow-ups. The EQ-5D-5 L evaluates health state, and the total score is indirectly calculated through a specifically developed equation. It is composed of five items (mobility, self-care, usual activities, pain, and anxiety/ depression). EQ-VAS uses a 100 mm line, which ranges from the worst health condition on the left end to the best health condition on the right end. Participants will be asked to mark the point corresponding to one's own health condition on that day.

Cost data investigation

Cost data include medical consumption, non-medical consumption and productivity loss cost of societal perspective. A questionnaire will be developed to evaluate consumption profiles, such as formal/informal medical consumption, non-medical consumption, and time consumption. While formal medical consumption refers to medical interventions and drugs, informal medical consumption refers to health food, medical instruments, etc. Non-medical consumption includes the cost of transportation, time consumption, and nursing. The Work Productivity and Activity Impairment questionnaire (WAPI) will be used for the cost of productivity impairment [24]. Medical consumption and non-medical consumption will be investigated with questionnaires which contains the cost data and the received treatment of participants and the cost data of Korean National Health Insurance will also be analyzed to calculate consumptions.

Credibility and expectancy questionnaire

The 9-point Likert scale for Credibility and Expectancy This scale is used to assess the participants' expectancy of the trial. On the 1st treatment visit, before the treatment, they will be asked how much the symptom would be reduced by the CMT and UC (1=not at all, 5=somewhat, and 9=very much).

Adverse events

Adverse events are undesirable and unintended signs (e.g., abnormal laboratory findings), symptoms, or diseases that occur after procedures during the clinical study, and they do not necessarily have to be causally related to the procedure. The analysis primarily includes adverse events suspected by the researcher to be related to the treatment, abnormal laboratory values, and the frequency of serious adverse events. Collected safety data are appropriately summarized, with all serious adverse events described narratively. Adverse events are collected through patient symptom reports and researcher observations and are identified by the frequency of adverse events between groups. The researcher will evaluate the causal relationship between each treatment method and the observed adverse events using a six-step scale (1 = definitely related, 2 = probably related, 3 = possibly related, 4 = probably not related, 5 = definitely not related, and 6 = unknown). All side effects will also be classified into three categories according to Spilker's classification method: Mild (1) - not requiring treatment and not significantly interfering with the subject's normal life (function); Moderate (2) - significantly interfering with the subject's normal life (function) and may require treatment, with recovery after treatment; Severe (3) - requiring intensive treatment due to severe adverse events and leaving sequelae.

6.3. Interpretation Methods (Statistical Analysis Methods)

6.3.1. Statistical Analysis

- This study is a randomized controlled trial of Chuna manual therapy for patients with temporomandibular joint pain, comparing the differences within and between groups based on effectiveness.

6.3.2 General principles of analysis

- This study analyzes the results of pain intensity scale, functional scale, quality of life, and cost data for patients who meet the inclusion criteria in a randomized controlled trial.
- The primary analysis will use the ITT (Intention-to-Treat) approach, evaluating all subjects who received at least one treatment. Additionally, the PP (Per-Protocol) analysis will be conducted alongside, evaluating only those subjects who completed the clinical study without dropping out. Missing data will be handled primarily through Multiple Imputation, and LOCF (Last Observation Carried Forward) will be used for sensitivity analysis.
- The socio-demographic characteristics and treatment expectancy of the study subjects will be evaluated by group. Continuous variables will be expressed as mean (standard deviation) or median (interquartile range) and compared between the two groups using Student's t-test. Categorical variables will be expressed as frequency (%) and analyzed using the chi-square test or Fisher's exact test.
- The efficacy evaluation variables of this clinical study are the differences in the changes of continuous outcomes (VAS, NRS, MMO, K-BDI-2, JFLS, SF-12, EQ-5D-5L, EQ-VAS) between the two groups from baseline to each time point. ANCOVA (Analysis of Covariance) will be performed with the baseline values of each variable and the covariate factors that show statistical differences between the

treatment groups at baseline as covariates, and the group as a fixed factor. RM ANOVA (Repeated Measures ANOVA) will be conducted to test the differences in trends at each visit.

- To compare the total amount of differences in each outcome within the period (evaluation time point after treatment completion (5 weeks)) and the entire study period (6 months), the areas under the curve (AUC) at each time point after randomization will be calculated and compared using Student's t-test.
- The proportion of patients whose NRS and VAS, indicators of temporomandibular joint pain, fall to less than half of the baseline will be compared and analyzed by time point. Kaplan–Meier survival analysis will be used to measure the time until the recovery of temporomandibular joint pain to less than half after randomization, and the curves will be compared using the log-rank test. A Cox model will be used to calculate the hazard ratio to compare the rate at which temporomandibular joint pain recovers to less than half, and it will also check if the recovery rate differs by subgroup.

- The significance level for all analyses is set at 0.05. All statistical analyses will be performed using the SAS version 9.1.3 statistical package (SAS Institute, Cary, NC, USA), with significance determined at $p<0.05$.

6.3.3. Economic evaluation
Economic evaluation will be implemented to compare the cost-effectiveness of CMT and UC. The primary economic endpoint will be an incremental cost-effectiveness ratio (ICER) of CMT compared with UC. ICER will be obtained by incremental cost between CMT and UC divided by incremental effectiveness between CMT and UC. Accordingly, additional cost will be calculated when 1 QALY is increased [26]. If predictions about subsequent periods are needed, cost and effectiveness after follow-up period will be extrapolated using regression models or secondary analysis such as decision modelling analysis. The treatment cost of this trial will be calculated by considering the number of treatments and their unit cost, derived from the health insurance cost and the agency cost. QOL inferred from the EQ-5D-5 L will be the primary measurement for the assessment of QALY, and the AUC method will be used [18]. If the total time horizon is more than 12 months, the cost unit will be standardized as the Korean currency (won), and a discount rate of 5% will be applied, based on the guidelines for economic assessment of the Health Insurance Review & Assessment Service. This analysis will be conducted from the societal perspective. Representative values (i.e. mean) of parameters will be used in the baseline analysis. Sensitivity analysis will be carried out in the form of probabilistic sensitivity analysis by using the distribution of all possible parameters and their representative values. In order to confirm the robustness of the analysis results, the probabilistic sensitivity analysis will be performed by setting the range of cost, effect and transition probability variable to 95% confidence interval. The utility weight and the transition probabilities will be analyzed with the triangular distribution and the cost data will be analyzed with the normal distribution for the distribution of the variable. Monte Carlo simulation will be performed 10,000 times and the mean and 95% confidence interval will be calculated. After conducting probabilistic sensitivity analysis, cost-effectiveness acceptability curve will be confirmed [27].

7. Safety Assessment Including Adverse Events

7.1. Adverse Event (AE)

1) Adverse Event (AE)
An adverse event refers to any undesirable and unintended sign (e.g., abnormal laboratory test results), symptom, or disease occurring after the procedure during a clinical study. It does not necessarily have to be causally related to the procedure.
2) Serious Adverse Event (SAE)
A serious adverse event during a clinical study of Chuna therapy is defined as an event that meets any of the following criteria:

- Results in death or is life-threatening
- Requires hospitalization or prolongation of existing hospitalization
- Causes persistent or significant disability or impairment
- Constitutes a medically important situation

Even if an event does not meet these criteria, if it is deemed to have a significant impact on the patient's safety and health, the responsible practitioner and related experts will decide whether it should be classified as a serious adverse event and take appropriate action.

7.2. Precautions for Use

Practitioners must monitor for adverse events before and after Chuna therapy and take prompt and appropriate measures if any adverse events occur.

7.2.1. Precautions During Chuna Therapy

- Anticipated general side effects, such as mobilization or stimulation of soft tissues around the joints following manual therapy, should be explained to the patient before treatment.
- When performing cervical Chuna therapy, (1) minimize the force of rotational techniques, (2) use techniques that induce segmental rotation with minimal vertebral artery pressure, and (3) avoid unnecessary stress on the vertebral artery and other structures to prevent excessive rotational load on the vertebral artery.
-

7.3. Assessment Criteria

Adverse events are assessed for their severity and causality using subjective and objective symptoms, physical examinations, and neurological evaluations as needed.

7.4. Assessment Methods

All anticipated phenomena (symptoms and signs, onset date, duration, etc.) resulting from adverse events during Chuna therapy must be recorded in the adverse event report form. Unrecorded events are classified as subjective symptoms. Adverse events are documented in separate case report forms to prevent exposure of patient assignment groups. The severity of symptoms is evaluated in stages based on the evaluation criteria. Causality with the procedure is assessed using the WHO-UMC causality scale, which classifies the relationship into six categories. The severity of subjective and objective symptoms is evaluated using Spilker's three-tier classification.

Spilker's Three-Tier Classification

- Mild (1): Does not require treatment and does not significantly impair the subject's normal life (function).
- Moderate (2): Significantly impairs the subject's normal life (function), may require treatment, and recovers after treatment.
- Severe (3): Requires intensive treatment due to severe adverse reactions, and may result in sequelae.

Causality with Procedure (WHO-UMC causality scale)

- Clearly related
- Likely related
- Possibly related
- Unlikely related
- Clearly not related
- Uncertain

The outcome of adverse event follow-up is recorded as:

- Recovered, no sequelae
- Recovered, with sequelae
- Recovering, adverse event persisting, no progression
- Not recovered, adverse event present, progressing
- Death
- Uncertain

Actions taken concerning the clinical study group and control group after adverse events are recorded as:

- Not applicable (does not affect the procedure)
- Temporary suspension of application
- Permanent suspension of application

Additional measures taken beyond planned treatment after adverse events are recorded as:

- None
- Medication
- Hospitalization/prolongation of hospitalization
- Therapeutic or diagnostic procedures
- Others

7.5. Interpretation Method
All adverse events reported during the study period are tabulated, and the incidence rate is calculated. The proportion of patients experiencing adverse events in each group is compared using Chi-square test or Fisher's Exact test.

7.6. Reporting Method
- Clinical researchers at each institution must explain all possible adverse events to co-researchers, subjects, or guardians and educate them to report any phenomena observed after the procedure. All local, systemic, or clinical pathology symptoms observed post-procedure must be recorded and stored in the case report form according to clinical research management standards. The principal investigator at each institution must describe and evaluate all symptoms during the clinical study in the clinical research result report. In the event of a "serious adverse event," it must be reported to the Institutional Review Board (IRB) and the principal clinical research institution (Kyung Hee University Korean Medicine Hospital) to determine whether to continue or discontinue the study.
- Additional safety information must be reported periodically until the adverse event is resolved (e.g., disappearance of the event or impossibility of follow-up). The principal investigator at each institution must conduct all aspects of the clinical study in accordance with the Declaration of Helsinki.

7.7. Reporting of Serious Adverse Events
- The clinical study principal investigator and personnel must ensure the safety of subjects and take immediate and appropriate action to minimize adverse events in the event of serious adverse events.
- Duties of each responsible party in the event of a "serious adverse event" during the clinical study:

1. **Principal Investigator's Duty** The principal investigator must immediately report the serious adverse event to the IRB and suspend part or all of the clinical study until further instructions are received.
2. **Clinical Researcher's Duty** Clinical researchers must immediately report any serious adverse events to the principal investigator and the IRB.
3. **IRB's Duty** The IRB must decide whether to continue or discontinue part or all of the clinical study in the event of a serious adverse event and issue necessary instructions, such as suspension, to the principal investigator.
4. **Sponsor's Duty** The sponsor must immediately notify all relevant institutions upon receiving reports of serious and unexpected adverse events from the principal investigators or clinical researchers.

8. Safety Monitoring Plan and Data Safety Monitoring Plan
Ensuring Subject Safety and Data Integrity: The plan includes a review of subject safety and a comparison of case report forms with source documents to ensure data completeness.

Monitoring Schedule: Monitoring will be conducted three times: an initial monitoring at the start of subject enrollment, one interim monitoring during the clinical study, and a final monitoring at the study's completion. The monitoring will be carried out by the monitoring staff of the Jaseng Medical Foundation Spine & Joint Research Institute.

9. Subject Information and Consent Forms
Individual Written Consent: All patients must provide written consent, which includes the purpose of the study, procedures, countermeasures in case of adverse events, and data privacy protection measures. The consent form provided as an appendix will be used.

Explanation and Consent Forms: The subject information and consent forms follow the templates provided in the appendix.

10. Measures for Subject Safety and Compensation Protocol for Victims
10.1. Confidentiality of Study Subjects
All personal information of study subjects will be strictly managed under the supervision of the Institutional Review Board (IRB). The confidentiality and protection of the personal information of study subjects will be maintained. All data collected from subjects who agree to participate in this study will be anonymized and may be provided to other institutions with anonymous codes for research purposes, excluding personal information.

10.2. Other Necessary Measures for Safe and Scientific Conduct of Clinical Studies
In case of direct injury related to the study, appropriate medical treatment will be provided as determined by the clinical study investigator, and compensation for damages will follow the pre-designated study-related insurance terms. Study subjects will be provided with an emergency contact number to reach the principal investigator or study staff if they have any questions, experience medical problems, or suffer study-related illnesses during the study period.

11. Recruitment of Study Subjects
Recruitment Methods: Study subjects will be recruited through press releases about the study, promotional posters inside and outside the clinical study institutions, and free magazine advertisements.

Promotional Posters: Promotional posters will follow the format provided in the appendix.

12. Case Report Forms
This study will follow the template of the 'Case Report Form for the Efficacy and Cost-Effectiveness Evaluation of Chuna Therapy for Temporomandibular Joint Disorders: A Randomized, Controlled, Multicenter Clinical Study' provided in the appendix.

13. Timeline
IRB Approval Plan Submission: June 2018
First Patient Treatment Start: After IRB approval
Last Patient Treatment Start: To be scheduled
Last Patient Follow-Up: To be scheduled
Data Collection Completion: To be scheduled
Analysis: To be scheduled
Report: To be scheduled

14. Ethical Considerations
14.1. Compliance with Laws and Regulations
This clinical study will be conducted in accordance with the Declaration of Helsinki and its latest amendments and/or all relevant regulations, following Good Clinical Practice (GCP) guidelines.

14.2. Informed Consent of Clinical Study Subjects
Before starting the clinical study, subjects will be thoroughly informed about the study's content and treatment methods (including efficacy, adverse reactions, and safety) and will be required to sign a consent form voluntarily. Only after obtaining the signed consent form can subjects participate in the clinical study, and a copy of the consent form will be provided to them.

14.3. Institutional Review Board (IRB)
IRB Submission: Before the start of the clinical study, the principal investigator must submit the clinical study protocol, informed consent form (ICF), and all relevant information to the IRB/EC for review and approval. All patient recruitment materials must also be approved by the IRB/EC.
Ongoing Reporting: The principal investigator must provide summary updates of the clinical study status to the IRB/EC annually or more frequently if required by the IRB/EC's conditions, policies, and procedures. Any protocol amendments must also be reported to the IRB/EC immediately.
Serious Adverse Event Reporting: Investigators must comply with the IRB/EC's requirements for reporting serious adverse events. Investigators may also receive other safety-related communications. They are responsible for ensuring that these reports are reviewed and processed in accordance with local health authority requirements and IRB/EC policies and procedures, and that they are kept in the clinical study site file.

14.4. Compensation for Injury
Refer to the attached compensation protocol for victims.

14.5. Confidentiality
Unique Identification Numbers: The sponsor will assign unique identification numbers to patients enrolled in the clinical study to maintain confidentiality. This means patient names will not be included in the data provided to the sponsor.
Medical Information Confidentiality: Patient medical information collected during this clinical study is

confidential and, except as permitted or required by law, will be disclosed to third parties only as allowed in the signed consent form (or separate authorization for the use and disclosure of personal health information).
Medical Information Disclosure: Medical information may be provided to appropriate medical personnel for treatment purposes.
Data Use: Data from this clinical study may be used for monitoring and investigation by the sponsor's monitors and representatives, and by the clinical study site's IRB/EC as required.

15. Management of Clinical Study Documents and Administrative Procedures

15.1. Data Storage and Disposal

A designated location for storing materials and records related to the conduct of the clinical study will be prepared to ensure security. After the completion of the final report, a custodian will be designated to store clinical study-related documents for three years from the study completion date. Copies of case report forms and other data collected during the study must be submitted to Kyung Hee University Korean Medicine Hospital within one month of study completion, while the originals will be stored separately according to the institution's IRB regulations. Records and documents will be shredded to prevent information leaks once the storage period has expired, and personal information will be destroyed in accordance with Article 16 of the Personal Information Protection Act Enforcement Decree.

15.2. Deviation from the Clinical Study Protocol

Investigators and staff must fully understand and strictly adhere to the protocol to prevent violations. To comply with the visit schedule, the staff must take appropriate actions, such as providing written notifications or conducting telephone monitoring for the next visit time. In case of unavoidable protocol violations, the following procedures will apply:

Serious Protocol Violations: For significant violations such as subject withdrawal/drop-out criteria, subject inclusion/exclusion criteria violations, failure to obtain consent, or mid-study drop-out criteria, subjects may be dropped from the study after consultation with the investigator.
Minor Violations: Minor violations must be accurately recorded along with the reasons and extent of the delay, and their impact on the clinical study will be considered during the result analysis.

15.3. Data Publication and Protection of Trade Secrets

The sponsor may provide information about the clinical study publicly to medical professionals and the public through conferences and publications, and must comply with all requirements for publishing clinical study results. Study results may not be published or used in part or whole without the consent of the principal institution, Kyung Hee University Korean Medicine Hospital.

15.4. Protocol Amendments

If protocol changes are necessary, the sponsor will prepare a protocol amendment. Except for changes required to eliminate immediate hazards to subjects or administrative changes, all amendments must receive IRB/EC approval before implementation.

Statistical Analysis Methods

1. Statistical Analysis

- This study is a randomized controlled trial of Chuna manual therapy for patients with temporomandibular joint pain, comparing the differences within and between groups based on effectiveness.

1.1 General principles of analysis

- This study analyzes the results of pain intensity scale, functional scale, quality of life, and cost data for patients who meet the inclusion criteria in a randomized controlled trial.
- The primary analysis will use the ITT (Intention-to-Treat) approach, evaluating all subjects who received at least one treatment. Additionally, the PP (Per-Protocol) analysis will be conducted alongside, evaluating only those subjects who completed the clinical study without dropping out. Missing data will be handled primarily through Multiple Imputation, and LOCF (Last Observation Carried Forward) will be used for sensitivity analysis.
- The socio-demographic characteristics and treatment expectancy of the study subjects will be evaluated by group. Continuous variables will be expressed as mean (standard deviation) or median (interquartile range) and compared between the two groups using Student's t-test. Categorical variables will be expressed as frequency (%) and analyzed using the chi-square test or Fisher's exact test.
- The efficacy evaluation variables of this clinical study are the differences in the changes of continuous outcomes (VAS, NRS, MMO, K-BDI-2, JFLS, SF-12, EQ-5D-5L, EQ-VAS) between the two groups from baseline to each time point. ANCOVA (Analysis of Covariance) will be performed with the baseline values of each variable and the covariate factors that show statistical differences between the treatment groups at baseline as covariates, and the group as a fixed factor. RM ANOVA (Repeated Measures ANOVA) will be conducted to test the differences in trends at each visit.
- To compare the total amount of differences in each outcome within the period (evaluation time point after treatment completion (5 weeks)) and the entire study period (6 months), the areas under the curve (AUC) at each time point after randomization will be calculated and compared using Student's t-test.
- The proportion of patients whose NRS and VAS, indicators of temporomandibular joint pain, fall to less than half of the baseline will be compared and analyzed by time point. Kaplan–Meier survival analysis will be used to measure the time until the recovery of temporomandibular joint pain to less than half after randomization, and the curves will be compared using the log-rank test. A Cox model will be used to calculate the hazard ratio to compare the rate at which temporomandibular joint pain recovers to less than half, and it will also check if the recovery rate differs by subgroup.

- The significance level for all analyses is set at 0.05. All statistical analyses will be performed using the SAS version 9.1.3 statistical package (SAS Institute, Cary, NC, USA), with significance determined at $p<0.05$.

1.2. Economic evaluation

Economic evaluation will be implemented to compare the cost-effectiveness of CMT and UC. The primary economic endpoint will be an incremental cost-effectiveness ratio (ICER) of CMT compared with UC. ICER will be obtained by incremental cost between CMT and UC divided by incremental effectiveness between CMT and UC. Accordingly, additional cost will be calculated when 1 QALY is increased [26]. If predictions about subsequent periods are needed, cost and effectiveness after follow-up period will be extrapolated using regression models or secondary analysis such as decision modelling analysis. The treatment cost of this trial will be calculated by considering the number of treatments and their unit cost, derived from the health insurance cost and the agency cost. QOL inferred from the EQ-5D-5 L will be the primary measurement for the assessment of QALY, and the AUC method will be used [18]. If the total time horizon is more than 12 months, the cost unit will be standardized as the Korean currency (won), and a discount rate of 5% will be applied, based on the guidelines for economic assessment of the Health Insurance Review & Assessment Service. This analysis will be conducted from the societal perspective. Representative values (i.e. mean) of parameters will be used in the baseline analysis. Sensitivity analysis will be carried out in the form of probabilistic sensitivity analysis by using the distribution of all possible parameters and their representative values. In order to confirm the robustness of the analysis results, the probabilistic sensitivity analysis will be performed by setting the range of cost, effect and transition probability variable to 95% confidence interval. The utility weight and the transition probabilities will be analyzed with the triangular distribution and the cost data will be analyzed with the normal distribution for the distribution of the

variable. Monte Carlo simulation will be performed 10,000 times and the mean and 95% confidence interval will be calculated. After conducting probabilistic sensitivity analysis, cost-effectiveness acceptability curve will be confirmed [27].
