# Supplementary material for: Effectiveness and cost-effectiveness of Chuna manual therapy for temporomandibular disorder: A randomized clinical trial
Source: PLoS One. 2025 May 7;20(5):e0322402. doi: 10.1371/journal.pone.0322402 (PMC12057850; doi:10.1371/journal.pone.0322402)
Supplement: Attachment — Submitted filename: Response to reviewers_241002.docx [file pone.0322402.s016.docx]

Journal requirements:

1. When submitting your revision, we need you to address these additional requirements.

Please ensure that your manuscript meets PLOS ONE's style requirements, including those for file naming. The PLOS ONE style templates can be found at
<https://journals.plos.org/plosone/s/file?id=wjVg/PLOSOne_formatting_sample_main_body.pdf> and
<https://journals.plos.org/plosone/s/file?id=ba62/PLOSOne_formatting_sample_title_authors_affiliations.pdf>.

-> I have checked your style requirements.

2. We note that the grant information you provided in the ‘Funding Information’ and ‘Financial Disclosure’ sections do not match.

When you resubmit, please ensure that you provide the correct grant numbers for the awards you received for your study in the ‘Funding Information’ section.

-> I have revised it.

3. Thank you for stating the following financial disclosure:
 [This research was funded by the Traditional Korean Medicine R&D Program through the Korea Health Industry Development Institute (KHIDI), which is funded by the Ministry of Health & Welfare, Republic of Korea (grant number HB16C0059).]. 
Please state what role the funders took in the study.  If the funders had no role, please state: ""The funders had no role in study design, data collection and analysis, decision to publish, or preparation of the manuscript.""
If this statement is not correct you must amend it as needed.
Please include this amended Role of Funder statement in your cover letter; we will change the online submission form on your behalf.

-> This research was funded by the Traditional Korean Medicine R&D Program through the Korea Health Industry Development Institute (KHIDI), which is funded by the Ministry of Health & Welfare, Republic of Korea (grant number HB16C0059) (JHC). The funders had no role in study design, data collection and analysis, decision to publish, or preparation of the manuscript.

4. We note that you have indicated that there are restrictions to data sharing for this study. PLOS only allows data to be available upon request if there are legal or ethical restrictions on sharing data publicly. For more information on unacceptable data access restrictions, please see <http://journals.plos.org/plosone/s/data-availability#loc-unacceptable-data-access-restrictions>.

Before we proceed with your manuscript, please address the following prompts:

a) If there are ethical or legal restrictions on sharing a de-identified data set, please explain them in detail (e.g., data contain potentially identifying or sensitive patient information, data are owned by a third-party organization, etc.) and who has imposed them (e.g., a Research Ethics Committee or Institutional Review Board, etc.). Please also provide contact information for a data access committee, ethics committee, or other institutional body to which data requests may be sent.

-> The data cannot be shared publicly because they contain potentially identifying information. Data are available upon request from Jung-hyun Kwon ([jhkwon0302@jaseng.co.kr](mailto:jhkwon0302@jaseng.co.kr)), the administrative officer of the Institutional Review Board (IRB) at Jaseng Hospital of Korean Medicine. Data will be provide to researchers who meet the criteria for access to confidential data, as determined by the IRB’s review and approval, and subsequently made available by the research team.

b) If there are no restrictions, please upload the minimal anonymized data set necessary to replicate your study findings to a stable, public repository and provide us with the relevant URLs, DOIs, or accession numbers. For a list of recommended repositories, please see
<https://journals.plos.org/plosone/s/recommended-repositories>. You also have the option of uploading the data as Supporting Information files, but we would recommend depositing data directly to a data repository if possible.

We will update your Data Availability statement on your behalf to reflect the information you provide.

5. For studies involving third-party data, we encourage authors to share any data specific to their analyses that they can legally distribute. PLOS recognizes, however, that authors may be using third-party data they do not have the rights to share. When third-party data cannot be publicly shared, authors must provide all information necessary for interested researchers to apply to gain access to the data. (<https://journals.plos.org/plosone/s/data-availability#loc-acceptable-data-access-restrictions>)

For any third-party data that the authors cannot legally distribute, they should include the following information in their Data Availability Statement upon submission:
1) A description of the data set and the third-party source
2) If applicable, verification of permission to use the data set
3) Confirmation of whether the authors received any special privileges in accessing the data that other researchers would not have
4) All necessary contact information others would need to apply to gain access to the data.

Reviewer #1: This article presents detailed data and analysis on the efficacy and cost-effectiveness of CMT for TMD. However, compared to the extensive outcome data, there needs to be more sufficient explanation and discussion. Unnecessary data should be excluded, or an explanation and discussion should be added to help the reader understand.

Additionally, I would like to suggest the following comments:

1. The abstract needs to include more information about the trial settings. For example, a sentence like “They underwent eight sessions of CMT and usual care for four weeks” without mentioning the group assignment confuses how the interventions and control were designed. We recommend adding information about the methods to the abstract and describing only the key points in the results section.

-> Thank you for your valuable feedback. We agree that the description of the trial settings needed clarification. We have revised the abstract to specify the random assignment of patients to the intervention and control groups and how the interventions were designed.

Patients were randomly assigned to either the CMT group, which underwent eight sessions of CMT over four weeks, or the usual care (UC) group, which received physical therapy for the same period, in a 1:1 ratio.

2. In the introduction, it is not appropriate to express the content of reference number 5 as “conservative treatments,” and the intervention applied in that study should be specifically expressed. In addition, the evidence for other treatments should not be determined based on only a few research results; more comprehensive evidence should be presented.

-> I appreciate your valuable comments. I have revised the manuscript as follows and added the references.

However, self-reported scores of patients’ satisfaction with nonsteroidal anti-inflammatory drugs, occlusal appliances and physical therapy were not significantly different from those of the no treatment group, indicating unsatisfactory treatment effects or improvement [5]. Thus, the interest in complementary and alternative medicine treatment modalities for TMDs, such as acupuncture or manual therapy, has been growing [6], with an increasing amount of evidence from research studies, including systematic review, supporting their effectiveness [7-9].

3. The method of CMT and physical therapy is not specifically presented. There should be a specific description that allows the reader to reproduce the procedure. In addition, since the treatment applied to each patient is different, a specific explanation of the criteria for why the treatment is applied differently to each patient is necessary.

- Thank you for comments. I have revised it as follows.
- These included three techniques for the TMJ (Sitting TMJ distraction with thumb technique, Sitting lateral pterygoid pushing with index finger technique, and Sitting TMJ manipulation with thumb technique) and three techniques for the cervical spine (Supine cervical spine distraction technique, Supine cervical spine JS distraction manipulation technique and Supine cervical spine manipulation technique). Korean medicine doctors (KMDs) administered one or more of the selected CMT techniques based on the patient’s condition and their clinical judgments.
- This study aimed to evaluated the effectiveness of CMT in a real-world setting, adopting a pragmatic approach. The choice of CMT techniques or types of physical therapy was determined by the physician based on the patients’ clinical condition.

4. The evidence for expressing the physical therapy in the control group as “commonly used methods” should also be described.

-> I have added the reference.

Abouelhuda AM, Khalifa AK, Kim Y-K, Hegazy SA. Non-invasive different modalities of treatment for temporomandibular disorders: review of literature. Journal of the Korean Association of Oral and Maxillofacial Surgeons. 2018;44(2):43.

5. The trial timeline needs to be clearly recognized. Please present the timeline in a figure or table and unify the expression of each time point in the manuscript.

-> I appreciated your valuable comments. I have added the table of timetable, which has been included as Table 1.

5. The manuscript's structure does not comply with CONSORT. Please appropriately reorganize the contents of 'Methods' and 'Results' using the headings and subheadings. In particular, please clearly distinguish between the contents on efficacy and cost-effectiveness.

-> I reorganized the subheadings in the Methods section to comply with CONSORT and I have distinguished between the contents on efficacy and cost-effectiveness.

6. Moving the baseline demographics in Table S2 into the main text seems appropriate.

-> Table S2 has been moved into the manuscript as Table 2. Thank you for your suggestion.

8. To increase visibility, I suggest organizing the results in Table 1 into separate tables by outcome measures.

-> I have revised the format of Table 1 (now changed to Table 3). Please review the updated Table 3.

9. The primary outcome measure for the research hypothesis and the other secondary outcome measures are not distinguished throughout the study. The results section should be described in separate paragraphs by outcome measures. In addition, in terms of conclusion, the results of the primary outcome measure should be presented first.

-> Thank you for your suggestion. The results section has been revised according to your recommendation.

At week 5 post-randomization, the decrease in the VAS scores for TMJ pain was non-significant between groups (Difference in decrease 5.96; 95% CI -1.49 to 13.42).

No significant differences were observed in NRS scores for pain and bothersomeness at week 5, 13, and 26, and similarly, no significant differences were found in VAS scores for TMJ pain.

However, functional outcomes improved significantly in the CMT group: JFLS-Global (0.59; 95% CI 0.13 to 1.05) and JFLS-Verbal and emotional (Difference in decrease 0.86; 95% CI 0.28 to 1.44) at week 5.

Quality of life measures also favored the CMT group: SF-12 PCS (-3.50; 95% CI -5.86 to -1.13) and EQ-VAS (-13.21; 95% CI -20.03 to -6.27) at week 5. By week 13, the SF-12 PCS continued to show improvement (-2.54; 95% CI -4.88 to -0.19) indicating sustained benefits.

The PGIC score showed significant improvement in the CMT group (-0.55; 95% CI -0.91 to -0.18) in week 5 and (-0.47; 95% CI -0.90 to -0.03) in week 13 (Table 3).

Additionally, the conclusion has been changed in both the abstract and in discussion.

The findings of this study suggest that, despite no significant differences in pain at week 5, CMT is a cost-effective and effective treatment option for patients with TMD, particularly for those who require improvements in function and quality of life.

Reviewer #2: The authors demonstrated the efficacy and cost-effectiveness of Chuna manual therapy compared to usual therapy in patients with temporomandibular disorder. This study has provided strengthened evidence for the clinical effectiveness and cost-effectiveness of Chuna manual therapy for these patients through randomized controlled trials, which have had limited evidence so far. However, some clarifications are needed to ensure the robustness of this evidence.

Major comments

First, there is a lack of explanation on how various pieces of information, such as clinical outcomes, utilities, and costs, are connected to the conclusions. For example, in this study, there was no significant difference in pain outcomes between Chuna manual therapy and usual care, yet the discussion highlights the reduction of pain as a strength of this study. A more logical explanation of this aspect should be provided. Additionally, the conclusion in the abstract states that Chuna manual therapy is a safer option compared to usual care, but it does not mention whether there is any statistical significance between the two groups.

- I apologize for the confusion caused by the mixed terminology in the discussion of fast recovery through survival analysis. The incorrect part mentioned in the discussion has been corrected with additional explanation. Furthermore, I have added the information regarding the lack of significant differences in AE occurrences under the safety subheading."

Secondly, while the paper includes a lot of information on clinical outcomes, utilities, and costs, it lacks detailed explanations for each piece of information, making it difficult to obtain sufficient and specific details. For example, it is unclear what treatments are included in Chuna manual therapy or usual care, and whether patients received more than one treatment. Although this can be found in the supplemental table, it is crucial for understanding the intervention and should be mentioned in the methods section. Additionally, while the QOL outcomes are presented, it is necessary to explain the score ranges of each tool and whether a higher score indicates a higher or lower QOL to properly understand the results.

- I fully agree your comments. As per your suggestions, I have added detailed information about the intervention in the methods section.

Thirdly, it is recommended to reorganize the sequence of the paper’s flow. The main points that the reader needs to know should be presented first, followed by the supporting evidence, to help the reader better understand the content.

- I have reorganized the method and the results section. I greatly appreciate your valuable comments.

Specific comments for each section

Abstract

1) Lines 35-36: Recommend including the WTP value.

-> I have added the WPT value ($26,375) in the abstract as per your suggestion.

Introduction

1) Lines 58-61: This part should be included in the methods section to explain the unit costs of CMT. Additionally, it is necessary to provide more details on why a cost-effectiveness study is needed (e.g., for reimbursement from NHI).

-> Thank you for your comment. We have revised the manuscript to move the explanation of the NHI coverage of Chuna Manual Therapy (CMT) to the Methods section, including details about the unit costs of CMT ($21–$54 depending on the technique). Additionally, we have expanded the rationale for the cost-effectiveness study, emphasizing that it is essential to support potential reimbursement for temporomandibular disorders (TMDs) under the NHI system.

In Korea, the national health insurance (NHI) coverage of CMT was implemented in 2019 [12]. The unit cost of CMT varies depending on the technique used, ranging from approximately $19 to $52. However, NHI reimbursement is currently limited to CMT for musculoskeletal diseases classified under the ICD-10 M code and injuries under the S code. Since TMDs are classified under the ICD-10 K07.6 code, CMT for these disorders is not covered by NHI. A cost-effectiveness study of CMT for TMDs is necessary to support the potential expansion of NHI reimbursement to include these disorders.

Methods

1) Lines 90-91: Which outcome was the assessor not involved in? Please clarify this. Additionally, since most QOL tools are measured by self-reported questionnaires, how is assessor blinding meaningful in this context?

-> Based on your comments, the following revisions have been made.

Outcome assessors were blinded to group allocation and were not involved in the treatment procedures. They were responsible only for assessing the outcomes, particularly the TMJ range of motion, including maximum mouth opening (MMO; pain-free).

2) Lines 94-96: What techniques of CMT were included in this RCT? Please provide a potential list of CMT techniques here, or the exact list if it has already been selected.

-> Thank you for your comments. I have added the lists of CMT techniques.

These included three techniques for the TMJ (Sitting TMJ distraction with thumb technique, Sitting lateral pterygoid pushing with index finger technique, and Sitting TMJ manipulation with thumb technique) and three techniques for the cervical spine (Supine cervical spine distraction technique, Supine cervical spine JS distraction manipulation technique and Supine cervical spine manipulation technique). Korean medicine doctors (KMDs) administered one or more of the selected CMT techniques based on the patient’s condition and their clinical judgments.

3) Lines 97-99: Please clarify whether a patient can receive only one treatment from the usual care list or more than one treatment from the list.

-> I apologize for the confusion caused by the error. I have clarified that a patient can receive one or more treatments from the usual care list.

For the usual care group, commonly used methods of physical therapy[15] for TMJ pain, including thermotherapy, ultrasound therapy, transcutaneous electrical nerve stimulation (TENS), and interferential current therapy (ICT), was administered, with the physician prescribing one or more therapies based on their judgement of the patient’s condition.

4) Line 101: Please mention the medications that were possibly administered to both groups.

-> I have added the following under the subheading ‘intervention’ in the methods section.

During the treatment period (up to 4 weeks), medications and treatments for TMD from other medical institutions were not allowed, but they were permitted and recorded during the follow-up period. Rescue medication such as acetaminophen was allowed for all participants during the treatment period,, with a maximum dosage of 4g per day, and its usage was recorded.

5) Line 103: Provide the rationale for why the primary outcome was assessed at 5 weeks rather than at a longer follow-up.

-> Thank you for your valuable comments. I have added it as follows.

In both the Chuna and usual care groups, pain assessments immediately following the interventions may not have been accurate due to intervention-induced sensitivity. Therefore, pain levels were measured one week after completing all assigned interventions, which was set as the primary endpoint

6) Lines 103-108: Provide detailed information about the outcome measures (e.g., range of scores, questionnaire composition, what higher scores mean) to understand the meaning of the scores.

-> I appreciate your comments. I have added detailed information about outcome measures as follows

Primary outcome

The primary outcome was the visual analog scale (VAS) score for the TMJ, which was assessed in week 5. In both the Chuna and usual care groups, pain assessments immediately following the interventions may not have been accurate due to intervention-induced sensitivity. Therefore, pain levels were measured one week after completing all assigned interventions, which was set as the primary endpoint. The VAS consisted of a 100-mm line, with one end representing no pain and the other representing the worst imaginable pain. Participants marked the line to indicate their TMD pain intensity over the past week. VAS scores for the TMJ were assessed at baseline, weeks 2, 3, 4, 5, 13 and 26.

Secondary Outcomes

NRS for TMJ pain & bothersomeness: The Numeric Rating Scale (NRS) was used to objectively assess subjective pain. Patients chose a number from 0 (no pain) to 10 (worst imaginable pain) to represent their discomfort since the last visit. NRS scores for pain and bothersomeness related to TMD were recorded at every visit, including baseline, weeks 5, 13 and 26.

Range of Motion (ROM) of the TMJ: ROM was evaluated at screening, baseline, weeks 2, 3, 4, 5, 13 and 26. Maximum mouth opening without pain, protrusion, and lateral movements were measured using a ROM ruler (Therabite, Sweden). Participants were instructed to open their mouths as widely as possible without pain, and to perform mandibular protrusion and lateral movements. These measurements followed the guidelines set by the international RDC/TMD consortium.

Korean version of Beck’s Depression Index-2 (K-BDI-2)[16]: This questionnaire was administered at baseline, week 5 and 26. It consists of 21 questions scored from 0 to 3, with higher scores indicating greater depression severity. A total score of 17 or higher indicated clinical depression, at which point participants were referred to a psychological clinic.

Jaw Functional Limitation Scale (JFLS) [17]: Participants completed this questionnaire at baseline and week 5. The questionnaire assessed jaw function in biting, moving, speaking, and emotional expression, using 20 items rated from 0 (no limitations) to 10 (maximum limitations).

Patient Global Impression of Change (PGIC): The PGIC was assessed week 5, 13 and 26. Participants rated their health improvement on a 7-point Likert scale, ranging from 1 (very much improved) to 7 (very much worse).

Short Form-12 Health Survey (SF-12), version 2 [18]: The SF-12 was completed at baseline, week 5, 13 and 26. This survey contains 12 items assessing health-related quality of life, with higher scores indicating better quality of life.

5-Level EuroQol-5 Dimension (EQ-5D-5L) and EuroQol Visual Analogue Scale (EQ-VAS): Both instruments were administered baseline, week 5, 13 and 26. The EQ-5D-5L evaluates health status across five dimensions (mobility, self-care, usual activities, pain, and anxiety/depression), while the EQ-VAS uses a 100-mm line to rate health, from worst to best possible condition.

Credibility and Expectancy Questionnaire: This 9-point Likert scale was used to measure participants' expectations for the trial’s outcome. The Credibility/Expectancy Questionnaire was administered prior to treatment, participants rated how much they expected their symptoms to improve from 1 (not at all) to 9 (very much).

Work Productivity and Activity Impairment (WPAI) Questionnaire[19]: The WPAI was used to assess productivity loss due to TMD. It measured absenteeism, presenteeism, and overall work impairment, as well as impairment in regular activities, for both employed and unemployed participants over the past week. The questionnaire was assessed at baseline, week 2, 3, 4, 13 and 26.

7) Lines 119-124: Which items were included in the costs (e.g., doctor’s visits)? Please add the list of items that were included in the cost estimate.

-> I have added the following based on your comments.

Considered costs included CMT, physical therapy, consultation for doctor visits, and X-rays. Additionally, any healthcare services utilized by patients during the follow-up period were also accounted for.

8) Lines 132-136: Recommend moving this section before the 'Unit Cost' section so that readers can understand the perspectives first and then learn how to calculate costs according to the two perspectives.

-> I have reorganized it based on your comments.

9) Lines 142-145: Please revise the sentence to correct the grammar. Also, clarify which covariates were added and the meaning of significance (e.g., significance level of 0.05 based on the p-value).

-> I apologize for the error. I have revised the sentence and comprehensive revisions have been made, particularly in the statistical analysis section, to address omissions and ensure accuracy.

Effectiveness was evaluated by analyzing changes from baseline at each time point for each group. ANCOVA was planned, using baseline values and any covariates showing statistical differences (p-value <0.05) between groups at baseline, with group as the fixed factor. However, no covariates showed significant differences between groups at baseline, so no covariates were included in the final analysis. The analysis was based on the intention-to-treat (ITT) principle, with missing data on costs and utilities handled using multiple imputation. Correlations between baseline covariates and outcomes informed the imputation model [20]. Baseline covariates included treatment allocation, sex, and age. Due to a skewed data distribution, imputations were performed using predictive mean matching and the Markov chain Monte Carlo method, generating 20 imputation sets with the mice package (version 3.6.0) in R version 4.0.1. For sensitivity analysis, a per-protocol (PP) analysis was conducted, including only participants who completed all assigned treatment sessions, with missing data addressed through multiple imputation.

AUCs were calculated from randomization to the final follow-up, and the cumulative outcome values of the two groups were compared using Student’s t-test. Furthermore, the proportion of patients (%) in each group was compared at each timepoint where VAS scores decreased to less than half of the baseline values. Kaplan–Meier curves were employed for survival analysis to compare the time until TMD pain ‘recovery’ was achieved, defined as pain outcomes decreasing to less than half of the baseline levels post-randomization, and the curves were analyzed using the log-rank test. Hazard ratios between the two groups were compared using the Cox proportional hazards model. A significance level of 0.05 was applied for this study, and all analyses were performed using SAS 9.4 (© SAS Institute, Inc., Cary, NC, USA) and R Studio 1.1.463 (© 2009–2018 RStudio, Inc.).

10) Lines 157-158: The results of the WTP were estimated from a publication in 2012, which was 12 years ago. Are they still applicable in 2024?

-> I have added it as an additional limitation in the discussion.

Moreover, a limitation of this study is that the WTP values used were based on a 2012 survey conducted in Korea, as no recent data were available.

Results

1) Lines 174-175: What do the scores of CMT 7.0 and usual care 6.5 refer to? Present the p-value indicating that there is no significant difference between the two groups.

-> I apologize for any confusion caused by the inaccurate expressions in the previous version of the manuscript.

No significant differences were found in sex, age, comorbidities, or RDC/TMD group distribution, or baseline scores for VAS, NRS, JFLS, K-BDI, EQ-5D-5L, EQ-VAS, and SF-12 between groups (Table 2). The Credibility/Expectancy Questionnaire (CMT: 7.0 ± 1.5; usual care: 6.5 ± 1.9, p=0.22) was also not statistically significant.

2) Lines 183-187: The method does not mention that medication can be used for both the intervention and comparator. Please add this information.

-> I apologize for the missing information. I have added it.

During the treatment period (up to 4 weeks), medications and treatments for TMD from other medical institutions were not allowed, but they were permitted and recorded during the follow-up period. Rescue medication such as acetaminophen was allowed for all participants during the treatment period,, with a maximum dosage of 4g per day, and its usage was recorded.

3) Lines 190-197: Since the method does not explain the scores for each outcome measure, it is difficult to understand the meaning of the direction and magnitude of each score. I recommend adding this information to both the methods section and the footnote of the table. Additionally, it is necessary to provide more explanation in the discussion to derive conclusions about the implications of the scores for various clinical outcomes.

-> I have added it to the methods section. Thank you for your valuable comments.

4) Lines 216-219: Please add the meaning of 'faster recovery' and specify what the survival analysis pertains to.

-> I greatly appreciated your valuable comments. I have added the survival analysis method in statistical analysis section.

Furthermore, the proportion of patients (%) in each group was compared at each timepoint where VAS scores decreased to less than half of the baseline values. Kaplan–Meier curves were employed for survival analysis to compare the time until TMD pain ‘recovery’ was achieved, defined as pain outcomes decreasing to less than half of the baseline levels post-randomization, and the curves were analyzed using the log-rank test. Hazard ratios between the two groups were compared using the Cox proportional hazards model.

- Additionally, I have clarified the meaning in the discussion.

Although the primary outcome of VAS scores at the 5-week point showed no significant difference, a survival analysis defining recovery as a more than 50% reduction in baseline pain demonstrated a hazard ratio of 1.75 (95% CI 1.01 to 3.03, p=0.047), indicating faster recovery in the CMT group.

5) Lines 248-251: Please use the correct unit for ICER ($/QALY) throughout the manuscript.

-> I have added it throughout the manuscript.

Discussion

1) Lines 307-309: Same comments as in Methods 1). The outcome measures include self-reported questionnaires. In this case, can we say that assessor-blinding is useful?

-> I have revised the limitation as follows.

Due to the study design, blinding of the physicians (KMDs) and participants was not possible, and outcome assessors were only blinded for specific outcomes, such as the TMJ range of motion, with some outcomes measured through patient-reported questionnaires.

2) Lines 275-280: Pain showed non-significant results, but function improved, and it is understood that the quality of life (QOL) improved as a result. Please provide a more detailed explanation of this by discussing the meaning of the QOL measures for each dimension.

-> I appreciate your valuable comments. I have added the following to the discussion based on your sugeestions.

Quality of life, measured by EQ-VAS and SF-12, showed significantly greater improvements in the CMT group. Chronic TMD impacts quality of life and social activities due to functional limitations caused by TMD [25, 26]. The significant improvement in quality of life, particularly in the SF-12 PCS score, may be attributed to enhanced functional recovery. Given that TMD affects essential activities like chewing and speaking, which influence social functioning and daily life, improvements in function likely contributed to the enhanced quality of life. With only mild adverse events comparable to the control group, CMT emerges as a safe and effective treatment option for TMD, particularly in improving function and quality of life.

3) Lines 291-292: In the results of this study, significant results for pain were not observed, yet there is a discrepancy with the reported pain improvement in the discussion. To maintain consistency between the study results and the discussion, it is necessary to thoroughly address this discrepancy.

-> I apologize for the confusion caused by the mixed terminology in the discussion of fast recovery through survival analysis. I have now clearly distinguished between fast recovery and pain, explicitly stating that there were no significant findings regarding pain.

4) Lines 300-302: Similar to the previous question, please explain the discrepancies regarding reducing pain in relation to the results of this study.

-> I have added as follows.

Unlike previous systematic reviews, this study did not observe significant differences in pain outcomes, which may be due to differences in the setting and control groups, as the systematic reviews included studies from China. This makes direct comparisons difficult and suggests the need for further research.

4) Lines 314-315: What is the meaning of a conservative treatment option? Also, Draw meaningful conclusions based on all the outcomes included in the results.

- Thank you for your valuable comments. I have revised the conclusion as follows.

The findings of this study suggest that, despite no significant differences in pain at week 5, CMT is a cost-effective and effective treatment option for patients with TMD, particularly for those who require improvements in function and quality of life. These results provide a basis for expanding national health insurance coverage of Chuna therapy on TMD in Korea.
