# Supplementary material for: Effectiveness and cost-effectiveness of Chuna manual therapy for temporomandibular disorder: A randomized clinical trial
Source: PLoS One. 2025 May 7;20(5):e0322402. doi: 10.1371/journal.pone.0322402 (PMC12057850; doi:10.1371/journal.pone.0322402)
Supplement: Attachment — Submitted filename: responsetoreviewers.docx [file pone.0322402.s017.docx]

Journal Requirements:

Please review your reference list to ensure that it is complete and correct. If you have cited papers that have been retracted, please include the rationale for doing so in the manuscript text, or remove these references and replace them with relevant current references. Any changes to the reference list should be mentioned in the rebuttal letter that accompanies your revised manuscript. If you need to cite a retracted article, indicate the article’s retracted status in the References list and also include a citation and full reference for the retraction notice.

- We have thoroughly checked the reference list and confirmed that there are no retracted references cited in the manuscript. However, in response to the reviewer's comments, we have added one additional reference, which resulted in updates to the reference numbering and citations throughout the manuscript.

[Note: HTML markup is below. Please do not edit.]

Reviewers' comments:

Reviewer's Responses to Questions

**Comments to the Author**

1. If the authors have adequately addressed your comments raised in a previous round of review and you feel that this manuscript is now acceptable for publication, you may indicate that here to bypass the “Comments to the Author” section, enter your conflict of interest statement in the “Confidential to Editor” section, and submit your "Accept" recommendation.

Reviewer #1: All comments have been addressed

Reviewer #3: (No Response)

2. Is the manuscript technically sound, and do the data support the conclusions?

The manuscript must describe a technically sound piece of scientific research with data that supports the conclusions. Experiments must have been conducted rigorously, with appropriate controls, replication, and sample sizes. The conclusions must be drawn appropriately based on the data presented.

Reviewer #1: Yes

Reviewer #3: Yes

3. Has the statistical analysis been performed appropriately and rigorously?

Reviewer #1: Yes

Reviewer #3: Yes

4. Have the authors made all data underlying the findings in their manuscript fully available?

The [PLOS Data policy](http://track.editorialmanager.com/CL0/http:%2F%2Fwww.plosone.org%2Fstatic%2Fpolicies.action%23sharing/1/010f01948e83130c-4c10824c-8277-431d-8712-c315ca1de26d-000000/5B5HqXA_80bEcKwJJLjuOaxa25Ikd18JndqazesCUTE=195) requires authors to make all data underlying the findings described in their manuscript fully available without restriction, with rare exception (please refer to the Data Availability Statement in the manuscript PDF file). The data should be provided as part of the manuscript or its supporting information, or deposited to a public repository. For example, in addition to summary statistics, the data points behind means, medians and variance measures should be available. If there are restrictions on publicly sharing data—e.g. participant privacy or use of data from a third party—those must be specified.

Reviewer #1: Yes

Reviewer #3: Yes

5. Is the manuscript presented in an intelligible fashion and written in standard English?

PLOS ONE does not copyedit accepted manuscripts, so the language in submitted articles must be clear, correct, and unambiguous. Any typographical or grammatical errors should be corrected at revision, so please note any specific errors here.

Reviewer #1: Yes

Reviewer #3: Yes

6. Review Comments to the Author

Please use the space provided to explain your answers to the questions above. You may also include additional comments for the author, including concerns about dual publication, research ethics, or publication ethics. (Please upload your review as an attachment if it exceeds 20,000 characters)

Reviewer #1: The manuscript has been appropriately revised overall, but some parts require supplementation. Here are some suggestions:

1. Revise the “Study timetable” table that appears twice.

- Thank you for your careful review and valuable feedback. We apologize for the oversight regarding the duplication of the “Study timetable” table. We have now corrected this by removing the redundant table. We appreciate your attention to detail and your constructive suggestions.

2. Please indicate the exact book “textbook of Chuna Manual Medicine” as a reference.

- Thank you for your valuable feedback. We have now explicitly cited the exact book, Textbook of Chuna Manual Medicine, as a reference in the manuscript. We appreciate your careful review and constructive suggestions.

3. “Efficacy” and “effectiveness” are used interchangeably throughout the manuscript. Considering the pragmatic study design, please consider whether it would be more appropriate to use “effectiveness” rather than “Efficacy.”

- Thank you for your insightful comment. We agree with your suggestion and have revised the manuscript to consistently use “effectiveness” instead of “efficacy,” considering the pragmatic study design. We appreciate your careful review and constructive feedback.

4. The criteria for treatment selection still need to be clarified. Six CMT techniques are presented, but the symptoms or criteria for each technique applied should be described in the methods or discussion part. The same explanation is required for physical therapy in the control group, and in particular, TENS and ICT were applied to different patients, so criteria for distinguishing them should be provided.

- Thank you for your valuable comment. We have revised the Methods section to clarify the criteria for treatment selection. Specifically, we have detailed the six CMT techniques and their corresponding indications based on the Textbook of Chuna Manual Medicine. These techniques were applied according to the patient's symptoms and functional impairments, with Korean Medicine Doctors (KMDs) selecting and documenting the applied techniques in electronic medical records and case report forms.
- Additionally, we have provided further clarification on the usual care group, specifying the selection criteria for physical therapy modalities, including Interferential Current Therapy (ICT) and Transcutaneous Electrical Nerve Stimulation (TENS). ICT was applied for deep muscle tension and myofascial stiffness, while TENS was prescribed for patients with pain-dominant symptoms. Thermotherapy and ultrasound therapy were used for general pain relief and muscle relaxation.
- For more details, please refer to the revised Methods section. We appreciate your constructive feedback, which has helped improve the clarity and precision of our manuscript.

5. Please indicate the statistically significant results in Table 3.

- Thank you for your suggestion. We have now indicated the statistically significant results in Table 3 using the following notation: ^a^ P < 0.001, ^b^ P < 0.01, ^c^ P < 0.05.

6. There was a significant improvement in some of the functional indices (JFLS, ROM) and quality of life indices (EQ-5D-5L, EQ-VAS, SF-12). However, expressing that the overall function and quality of life improved is not appropriate. It is necessary to describe exactly which functions and quality of life improved in a limited way.

- Thank you for your valuable feedback. In response to your comment, we have revised the manuscript to specify the functional and quality of life measures that showed significant improvements rather than making a generalized statement about overall function and quality of life.
- In the **abstract**, we now state:
  "The CMT group showed significant improvement in specific functional and quality of life measures, particularly in the EuroQoL-VAS (-13.21 (95% confidence interval [CI] -20.03 to -6.38) and the Jaw Functional Limitation Scale-Global score of 0.59 (95% CI 0.13 to 1.05), though improvements were not consistent across all indices."
- In the **Discussion section**, we have clarified:
  "Functional outcomes also improved significantly, particularly in JFLS-Global (0.59, 95% CI 0.13 to 1.05) and JFLS-Verbal and Emotional (0.86, 95% CI 0.28 to 1.44). These improvements in function may have contributed to the observed enhancements in quality of life, as measured by EQ-VAS and SF-12 PCS, in the CMT group."
- Additionally, in the **Limitations section**, we have explicitly noted:
  "Moreover, although quality of life and functional improvements were observed, significant effects were found only in specific measures, such as EQ-VAS, SF-12 PCS, and JFLS-Global/Verbal and Emotional scores, rather than across all assessed indices. This suggests that the impact of CMT on overall function and quality of life may not be uniform across all measures."
- These revisions ensure that our conclusions accurately reflect the findings without overstating the results. We appreciate your constructive comments, which have helped improve the precision and clarity of our manuscript.

7. The “7.3±0” value in the S2 Table seems to be an error.

- Thank you for pointing this out. We have corrected the value in **Supplementary Table 2** from **7.3±0** to **7.3±1.2**.

Reviewer #3: This review concentrates primarily on the statistical analysis. In general, the analysis seems sound. However, the statistical methods section should be rewritten slightly to clarify the various components of the statistical analysis.

Was the AUC used as a way of time-averaging the endpoint(s)? Please motivate the use of the AUC in this setting.

- The Area Under the Curve (AUC) analysis was employed in this study as a method to comprehensively capture the temporal changes in outcomes throughout the entire study period. Rather than simply analyzing isolated timepoints, the AUC approach cumulatively reflected the total amount of effectiveness outcomes from randomization to the final follow-up. This methodology provides a more holistic evaluation of treatment effects over time by considering the entire trajectory of patient response.

More generally, note that each study assessment gives rise to one or more endpoints, which are used to assess the hypotheses of the study. With this in mind, please indicate the statistical methods section which endpoints are assessed via which statistical methods, as well as any transformations that are applied before statistical analysis (e.g., the AUC). This should help clarify the flow from data to results to conclusions.

Following your advice, we changed the statistical analysis section as follows:

- During the study period, AUC analysis and survival analysis were conducted to comprehensively capture the temporal changes in outcomes. The AUC cumulatively reflected the total amount of effectiveness outcomes from randomization to the final follow-up and was calculated on a 1-week basis according to the trapezoidal rule. The AUC of the two groups were compared using Student's t-test. Furthermore, the proportion of patients (%) in each group was compared at each timepoint where VAS scores decreased to less than half of the baseline values. Kaplan–Meier curves were employed for survival analysis to compare the time until TMD pain ‘recovery’ was achieved, defined as pain outcomes decreasing to less than half of the baseline levels post-randomization, and the curves were analyzed using the log-rank test. Hazard ratios between the two groups were compared using the Cox proportional hazards model. A significance level of 0.05 was applied for this study, and all analyses were performed using SAS 9.4 (© SAS Institute, Inc., Cary, NC, USA) and R Studio 1.1.463 (© 2009–2018 RStudio, Inc.).

To further elaborate, generally, the structure should be rather parallel, with:

Hypotheses -> Assessments -> Endpoints -> Statistical methods -> Results -> Conclusions

- Thank you for your valuable suggestion. To ensure clarity and coherence in the manuscript, we have explicitly incorporated the study hypothesis within the Study Design subsection.

**Study design**

This study was designed as a two-arm, multicenter, assessor-blinded RCT to evaluate the effectiveness and cost-effectiveness of CMT compared to usual care in patients with myofascial TMD. We hypothesized that CMT would result in greater pain reduction, improved jaw function, enhanced health-related quality of life, and favorable cost-effectiveness profile. Additionally, we hypothesized that CMT would lead to faster symptom relief and recovery compared to usual care.

This study was conducted from September 24, 2018, to June 29, 2019, at one university hospital (Kyung Hee University Korean Medicine Hospital, Gangdong) and four spine specialty Korean medicine hospitals (Jaseng Hospital of Korean Medicine in Gangnam, Daejeon, Bucheon, and Haeundae). The protocol of this RCT has been published [14] and was approved by the Institutional Review Board of the respective hospitals (JASENG 2018-06-008, 2018-06-010, 2018-06-011, and 2018-06-012, and KHNMCOH 2018-05-007). Written informed consent was obtained from all participants. This study was conducted according to the Consolidated Standards of Reporting Trials. The study timetable is presented in Table 1.

It would be useful to have figures that display longitudinal data --- these could be included just as supplementary figures.

- Thank you for your helpful suggestion. We have added **S2 Figure** to display the longitudinal data as recommended. Additionally, we have revised the main text to reflect this addition, stating:

Longitudinal changes in outcomes, including pain(VAS), functional measures (JFLS-Global), and quality of life indicators (SF-12 PCS, EQ-VAS, EQ-5D), are visualized in S2 Figure.

7. PLOS authors have the option to publish the peer review history of their article ([what does this mean?](https://track.editorialmanager.com/CL0/https:%2F%2Fjournals.plos.org%2Fplosone%2Fs%2Feditorial-and-peer-review-process%23loc-peer-review-history/1/010f01948e83130c-4c10824c-8277-431d-8712-c315ca1de26d-000000/AyBCZBe8iB_H1LYZp3fG864xkwuU5NrhUH_a-BLPDYY=195)). If published, this will include your full peer review and any attached files.


If you choose “no”, your identity will remain anonymous but your review may still be made public.


**Do you want your identity to be public for this peer review?** For information about this choice, including consent withdrawal, please see our [Privacy Policy](https://track.editorialmanager.com/CL0/https:%2F%2Fwww.plos.org%2Fprivacy-policy/1/010f01948e83130c-4c10824c-8277-431d-8712-c315ca1de26d-000000/uxBs7j3gqSol8qBmz1A7_dea8ozfBqSMYNj0uVDdIes=195).

Reviewer #1: No

Reviewer #3: No
